# Synergistically Boosting the Circularly Polarized Luminescence of Functionalized Pillar[5]arenes by Polymerization and Aggregation

**DOI:** 10.1002/advs.202305149

**Published:** 2023-10-22

**Authors:** Hewei Yan, Xiaojun Yin, Dong Wang, Ting Han, Ben Zhong Tang

**Affiliations:** ^1^ Center for AIE Research, Shenzhen Key Laboratory of Polymer Science and Technology, Guangdong Research Center for Interfacial Engineering of Functional Materials, College of Materials Science and Engineering Shenzhen University Shenzhen Guangdong 518060 China; ^2^ College of Physics and Optoelectronic Engineering Shenzhen University Shenzhen 518060 China; ^3^ School of Science and Engineering, Shenzhen Institute of Aggregate Science and Technology The Chinese University of Hong Kong Shenzhen (CUHK‐Shenzhen) Guangdong 518172 China

**Keywords:** aggregation‐induced emission, circularly polarized luminescence, pillar[n]arene, intramolecular motions, planar chirality

## Abstract

Supramolecular polymers based on chiral macrocycles have attracted increasing attention in the field of circularly polarized luminescence (CPL) owing to their unique properties. However, the construction of macrocyclic supramolecular polymers with highly efficient CPL properties in aggregate states still remains challenging. Herein, w e constructed a class of macrocycle‐based coordination polymers by combining the planar chiral properties of pillar[5]arene with the excellent fluorescence properties of aggregation‐induced emission luminogens. The formation of polymers enhances both the fluorescence and chiral properties, resulting in chiral supramolecular polymers with remarkable CPL properties. Increasing the aggregation degree of the polymers can further improve their CPL properties, as evidenced by a 21‐fold increase in the dissymmetry factor and an over 25‐fold increase in the fluorescence quantum yield in the aggregate state compared to the solution state. Such a synergistic effect of polymerization‐ and aggregation‐enhanced CPL can be explained by the restriction of intramolecular motions and aggregation‐induced conformation confinement. This work provides a promising method for developing highly efficient CPL supramolecular polymers.

## Graphical abstract

*A novel class of circularly polarized luminescence (CPL)‐active macrocycle‐based coordination polymers are constructed taking advantage of the planar chiral properties of pillar[5]arene and the excellent fluorescence properties of aggregation‐induced emission luminogens. Remarkably boosted CPL is achieved through the synergistic effects of polymerization and aggregation processes based on the restriction of intramolecular motions and aggregation‐induced conformation confinement.

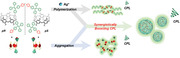

## 1 Introdution

Circularly polarized luminescence (CPL) is a type of light emission that contains the differential emission intensity of left‐ and right‐handed circularly polarized light, which can provide information about the structural and electronic properties of chiral luminescent systems in the excited state. Functional materials with CPL have shown promising prospects in diverse high‐tech applications such as th displays, information storage, intelligent detection, optoelectronic devices, asymmetric synthesis, contrast imaging, and other fields.^[^ ^1^, ^2^, ^3^, ^4^, ^5^, ^6^ ^]^ Compared to traditional physical methods such as the use of linear polarizers and quarter‐wave plate, the direct generation of CPL based on the rational design of chiral luminophores effectively avoids the energy loss during the inter‐plate transition and has gradually become the mainstream method for studying CPL.^[^ ^7^ ^]^ Chirality and luminescence are two preconditions for constructing CPL materials, in which the luminescence dissymmetry factor (*g*_lum_) and fluorescence quantum yield (*Φ*) are two critical parameters for evaluating the performance of CPL materials.^[^ ^8^, ^9^ ^]^ To meet the requirements of practical applications and meanwhile to enrich the structural diversity of CPL emitters, it is of great significance to design and construct novel CPL materials with efficient *g*_lum_ and high *Φ*.

Among various chiral materials, macrocycle‐based chiral emitters have received increasing attention due to their unique structural features and excellent chiral properties.^[^ ^10^, ^11^, ^12^, ^13^ ^]^ As an important group of macrocyclic chiral molecules, pillar[*n*]arenes with easily functionalized cylindrical structures and outstanding host‐guest binding ability have been widely used in drug delivery, chemical sensing, separation and purification, biomedical applications, etc.^[^ ^14^, ^15^, ^16^, ^17^ ^]^ Typically, pillar[*n*]arenes are racemic with two planar chiral isomers *pS*:*pR* = 1:1, since these two isomers could interconvert between each other via active oxygen‐through‐the‐annulus rotations of their hydroquinone rings.^[^ ^18^ ^]^ By guest complexation, environmental regulation, hydrogen bonding formation, or introducing bulky substituents to the rim, such intramolecular rotations can be effectively restricted to lock the planar chirality of pillar[*n*]arenes.^[^ ^19^, ^20^, ^21^, ^22^, ^23^, ^24^ ^]^ The introduction of luminophores with large steric hinderance can endow the modified pillar[*n*]arenes with both locked planar chirality and luminescent properties, thus making them good building blocks for the construction of CPL materials.^[^ ^25^, ^26^ ^]^ Despite of the rapid development of small molecular chiral pillar[*n*]arenes, CPL‐active supramolecular polymer systems based on pillar[*n*]arenes have been rarely investigated. Compared to the small molecular counterparts, the inherently complicated and multidimensional structures as well as the dynamic and reversible non‐covalent interactions of supramolecular polymers could facilitate the chiral amplification^[^ ^27^, ^28^ ^]^ and fluorescence modulation of pillar[*n*]arene‐based emitters,^[^ ^12^, ^13^, ^29^, ^30^ ^]^ thereby providing new possibilities for the development of high‐performance CPL materials. Attracted by these merits, we thus aim to developing pillar[*n*]arene‐based chiral supramolecular polymers with efficient CPL properties.

Till now, a wide variety of pillar[*n*]arene‐based fluorescent supramolecular polymers have been developed by introducing fluorescent moieties to the host (pillar[*n*]arene) or the guest structures and then forming polymers through self‐assembly or host‐guest interactions.^[^ ^25^, ^26^, ^29^, ^30^, ^31^, ^32^, ^33^ ^]^ However, the luminescence performance of traditional planar luminophores tends to be diminished or even completely annihilated upon aggregation due to the presence of strong π‐π stacking interactions.^[^ ^34^, ^35^, ^36^ ^]^ This aggregation‐caused quenching effect greatly compromises the CPL performance of the corresponding supramolecular polymer materials in aggregate states and consequently limits their application scope. In practical applications such as security tags, biomedical applications in aqueous media, and photoelectric devices, CPL materials are preferred to be used as aggregate forms like nanoparticles and solid thin films. Therefore, it is highly desirable to develop chiral supramolecular polymers with efficient aggregate‐state luminescence. Unlike traditional light emitters, luminogens with aggregation‐induced emission (AIEgens) such as tetraphenylethene (TPE) are weakly emissive in dispersed states but become highly luminescent in aggregate states.^[^ ^37^, ^38^, ^39^ ^]^ Besides the constructive role of aggregation process, the restriction of intramolecular motions (RIM) in dispersed states can also enhance the photoluminescence (PL) performance of AIEgens to show high *Φ* because of the suppressed non‐radiative decay pathway of excitons.^[^ ^40^, ^41^ ^]^

**Figure 1:**
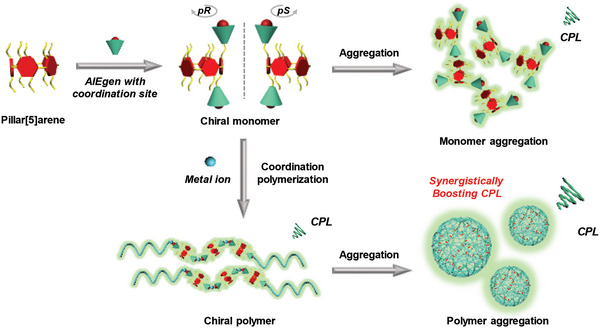
Schematic illustration of the design strategy for the construction of pillar[5]arene‐based materials with polymerization‐ and aggregation‐enhanced circularly polarized luminescence (CPL).

Considering the positive effects of RIM on both the planar chirality locking of pillar[*n*]arenes and fluorescence enhancement of AIEgens, herein we explored the possibility of constructing chiral supramolecular polymers with efficient CPL performance in aggregate states based on AIEgen‐modified pillar[5]arene monomers. An effective strategy for boosting the CPL performance of functionalized pillar[5]arenes was proposed based on the synergistically strengthened RIM via polymerization and aggregation. As illustrated in **Figure** **1**, an AIEgen with a metal coordination site was employed as the modification group to partially lock the planar chirality of pillar[5]arene and meanwhile endow the material with AIE features. Supramolecular coordination polymers were further formed by coordinating the AIEgen‐modified pillar[5]arene monomer with metal ions via the introduced chelation sites.^[^ ^42^, ^43^ ^]^ In addition to the inherent RIM effect of the AIEgen substituent, the coordination polymerization process could further rigidify the molecular conformations and suppressed the intramolecular motions, consequently leading to the enhanced CPL performance by simultaneously boosting the chiral and PL properties. Thanks to the presence of AIEgens, the occurrence of physical aggregation facilitates the further enhancement in the CPL performance of the chiral monomers and supramolecular polymers. The synergistic effect of polymerization‐ and aggregation‐enhanced CPL provides a simple and feasible way to achieve high‐performance CPL‐active supramolecular polymer systems in aggregate states.

## 2 Results and Discussion

**Figure 2:**
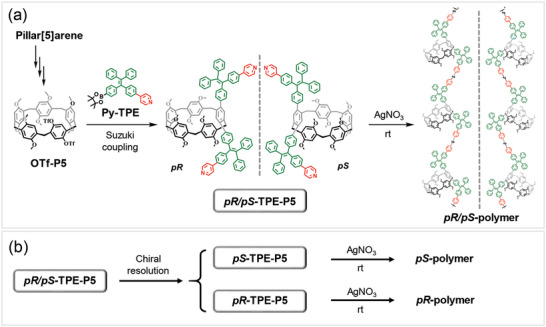
Chemical structures and synthetic routes of the AIEgen‐functionalized pillar[5]arenes and the corresponding coordination supramolecular polymers.

To satisfy the aforementioned function requirements, we designed and synthesized a pyridine‐conjugated TPE borate ester (Py‐TPE) and covalently introduced it to the two sides of pillar[5]arene rims as an AIE‐active substituent with large steric hindrance (**Figure** **2**). As depicted in Figure S1, firstly, a reactive pillar[5]arene (OTf‐P5) was prepared through the selective and sequential oxidation and reduction reactions of pillar[5]arene followed by the protection reaction with benzenesulfonic acid according to the previously reported protocols.^[^ ^11^, ^25^, ^44^ ^]^ Then Py‐TPE was prepared by introducing pyridine (metal ion coordination site) and borate ester (reactive site) into 1,1‐diphenyl‐2,2‐di(p‐bromophenyl)ethylene. To avoid the formation of stereoisomers of difunctionalized TPE, the pyridine unit and borate ester group were placed at the two benzene rings connected to the same carbon atom in TPE skeleton. Finally, the target compound *pR*/*pS*‐TPE‐P5 was produced as a mixture of two planar chiral isomers by the Suzuki coupling reaction between OTf‐P5 and Py‐TPE under the catalysis of tetrakis(triphenylphosphine)palladium. Through further isolation via high performance liquid chromatography (HPLC) equipped with a Daicel Chiralpak IG column, a pair of optically pure enantiomers, namely *pR*‐TPE‐P5 and *pS*‐TPE‐P5, were successfully obtained. With these dual‐ligand macrocyclic monomers (*pR*‐TPE‐P5, *pS*‐TPE‐P5 and *pR*/*pS*‐TPE‐P5) in hand, we next conducted the coordination polymerizations via the coordination interactions between the pyridine ligands and silver ions to afford three supramolecular polymers named as *pR*‐polymer, *pS*‐polymer and *pR*/*pS*‐polymer, respectively.

**Figure 3:**
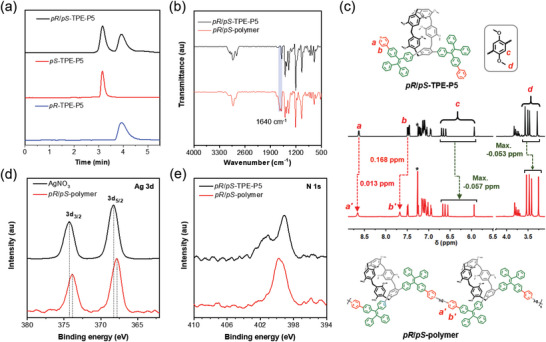
Structural characterization of the target pillar[5]arene‐based monomers and supramolecular polymers: a) HPLC chromatograms of *pR*/*pS*‐TPE‐P5, *pS*‐TPE‐P5 and *pR*‐TPE‐P5 recorded by a chiral HPLC equipped with a Daicel Chiralpak IG column using DCM/MeOH mixture (V/V = 1/1) as the eluent. b) Comparison between the IR spectrum of *pR*/*pS*‐TPE‐P5 and that of the *pR*/*pS*‐polymer. c) ^1^H NMR spectra of *pR*/*pS*‐TPE‐P5 and *pR*/*pS*‐polymer. d) and e) High‐resolution X‐ray photoelectron spectra of the Ag 3d and N 1s of *pR*/*pS*‐TPE‐P5 and *pR*/*pS*‐polymer.

The structure of the target compound (*pR*/*pS*‐TPE‐P5) was fully characterized and confirmed by ^1^H NMR, ^13^C NMR and HRMS spectrometry, and the involved intermediate and optically pure enantiomer structures were also carefully characterized and verified (Figures S2‒S12). Due to the presence of bulky Py‐TPE substituents on the rims, the intramolecular rotations of pillar[5]arene skeleton are restricted to some extent. Therefore, the interconversion between the two TPE‐P5 enantiomers could be partially suppressed to enable the chiral resolution. As shown in **Figure** **3a** and Figure S10, the initial injection of *pR*/*pS*‐TPE‐P5 into chiral HPLC analysis resulted in two separated peaks with 2.2% ee, indicating that the mixture of *pR*/*pS*‐TPE‐P5 is indeed not racemic. When the isolated fraction was injected into the chiral HPLC system, only one single peak was observed in the chromatogram, suggesting that the planar chirality of *pR‐* and *pS*‐TPE‐P5 is stable. The formation of coordination polymers was confirmed by a series of characterization techniques (Figures S13‒S20). Taking the *pR*/*pS*‐polymer as an example, compared with the IR spectrum *pR*/*pS*‐TPE‐P5 monomer, the additional peak at 1640 cm^−1^ in the IR spectrum of *pR*/*pS*‐polymer could be attributed to the stretching vibration of the coordinated pyridyl group (**Figure** **3b**).^[^ ^45^ ^]^ Regarding the NMR results, the resonance peaks related to the pyridyl protons at “a” and “b” positions of *pR*/*pS*‐TPE‐P5 shifted to the downfield after the coordination intereaction with silver ions, while some of the aromatic and OCH_3_ protons on pillar[5]arenes shifted upfield by 0.057 ppm in the ^1^H NMR spectrum of *pR*/*pS*‐polymer (**Figure** **3c** and Figures S14). The other resonance peaks associated with the aromatic protons of TPE moiety in the range of −0.035 to 0.004 ppm also varied after reaction. Similar changes were also observed in the IR and ^1^H NMR spectra of *pR*‐polymer and *pS*‐polymer (Figure S15‒S19). The X‐ray photoelectron spectroscopy (XPS) results further supported the occurrence of the supramolecular coordination polymerization between the pyridine‐modified macrocyclic monomer and silver ions. As shown in Figure S20, new peaks related to Ag element appeared in the XPS spectrum *pR*/*pS*‐polymer. Close inspection on the Ag 3d XPS signal reveals that the binding energies of Ag element in the polymer slightly decrease than those in silver nitrate (**Figure** **3d**). Meanwhile, the high‐resolution N 1s spectrum of *pR*/*pS*‐polymer also shows obvious different binding energies and lineshape compared with that of *pR*/*pS*‐TPE‐P5 (**Figure** **3e**). The abovementioned results collectively demonstrated the successful preparation of these three supramolecular coordination polymers.

**Figure 4:**
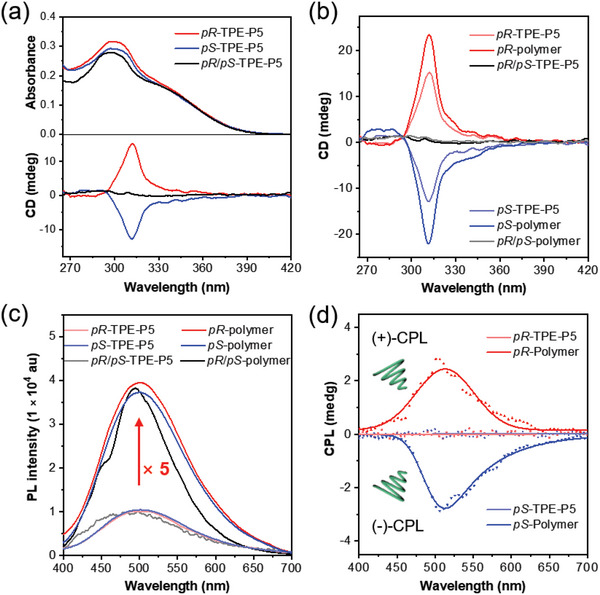
Polymerization‐enhanced CPL: a) The UV‐vis and CD spectra of the pillar[5]arene‐based monomers in DMSO solutions. Concentration: 10 µM. b) The CD spectra of the pillar[5]arene‐based monomers and supramolecular polymers in DMSO solutions. Concentration: 10 µM. c) Photoluminescence (PL) spectra of the pillar[5]arene‐based monomers and supramolecular polymers in DMSO solutions. Concentration: 10 µM. d) CPL spectra of *pR*‐TPE‐P5, *pS*‐TPE‐P5, *pR*‐polymer, and *pS*‐polymer in solution state. Concentration: 10 µM. The scattered data represents the raw data and the solid lines are the nonlinear fitting curves obtained by the Gaussian algorithm.

We next investigated the chiroptical and photophysical properties of the obtained AIEgen‐functionalized pillar[5]arene monomers and the coordination supramolecular polymers in dilute solutions. As shown in **Figure** **4a**, *pR*/*pS*‐TPE‐P5 and the isolated enantiomers possessed similar absorption properties with the maximum absorption wavelength at 298 nm, whereas their circular dichroism (CD) spectra showed remarkable difference. Negligible CD signal was detected from the DMSO solution (10 µM) of the unseparated mixture (*pR*/*pS*‐TPE‐P5), while a pair of mirror images appeared in the CD spectra of the isolated enantiomers, suggesting that the configurations of both enantiomers could be sufficiently maintained by the bulky and rigid π‐conjugated substituents.^[^ ^20^, ^26^ ^]^ The comparison between the experimental CD spectra (Figure 4a) and the theoretically simulated CD spectra (Figure S21) further verified the absolute configurations of the isolated fractions. The peaks at the retention time of 3.16 and 3.95 min of the fractions in chiral HPLC analysis (Figure 3a) can be assigned as *pS*‐TPE‐P5 and *pR*‐TPE‐P5, respectively. The isolated enantiomer *pR*‐TPE‐P5 showed positive CD signal while *pS*‐TPE‐P5 depicted negative CD signal. The chiroptical properties of the polymers were then analyzed and compared with those of the corresponding monomers. The absorption spectra (Figure S22) and CD spectra of the obtained pillar[5]arene‐based supramolecular polymers peaked at similar wavelength to their monomers, but the CD intensity of the coordination polymers was obviously stronger than that of the monomers under the same solution concentration (**Figure** **4b**). The CD signal of *pR*‐polymer at 312 nm increased from 15.2 to 23.5 mdeg, while the negative CD intensity of *pS*‐polymer enhanced from −12.8 to −22.0 mdeg. Such a chirality amplification phenomenon was speculated to arise from the collective effects

of larger steric hindrance and multiple intra‐/intermolecular interactions in supramolecular polymers.^[46−49]^ On the one hand, the steric hindrance at both ends of pillar[5]arenes increased after polymerization, and the strengthened RIM effect was conducive to the chirality fixation of the polymer. On the other hand, the structure of supramolecular polymer is inherently more complex than that of small molecules. The multidimensional structure and interchain entanglement caused by multiple interactions can also promote the chiral amplification. Further investigation on the PL spectra also supports our speculation. As summarized in **Figure** **4c**, the PL intensity of *pR*‐ and *pS*‐polymer was about five times higher than that of *pR*‐ and *pS*‐TPE‐P5 at the same concentration with the maximum emission wavelength locating at ∼500 nm. The *Φ* values of coordination polymers were measured to be 1.8%‒2.0% in dilute solutions, which were also enhanced than their monomers (*Φ* = 0.9%‒1.0%, Table S1). As expected, the simultaneous enhancement of chirality and PL intensity greatly facilitated the generation of CPL as evidenced by the obvious CPL signals of *pR*‐polymer and *pS*‐polymer with an average |*g*_lum_| of 1.30 × 10^−4^ (**Figure** **4d** and Figures S23‒S24). By contrast, the solutions of AIEgen‐functionalized pillar[5]arene monomers (*pR*‐TPE‐P5 and *pS*‐TPE‐P5) showed faint CPL signals despite of their good CD properties. These results implied that the fluorescence efficiency may play a critical role in the achievement of efficient CPL.

**Figure 5:**
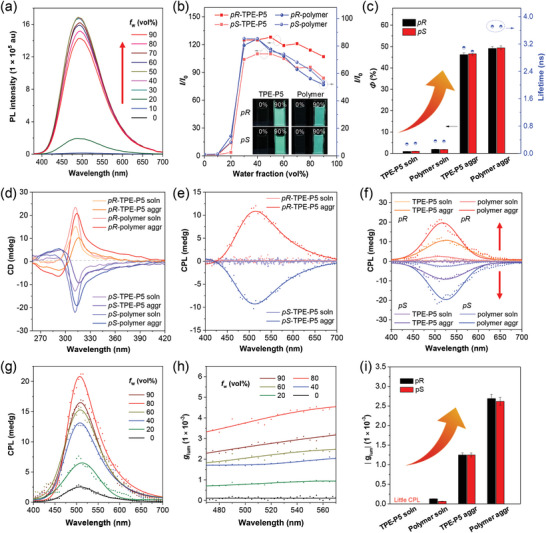
Polymerization and aggregation synergistically enhanced CPL: a) Photoluminescence (PL) spectra of *pR*‐TPE‐P5 in DMSO/water mixtures with different water fractions (*f*_w_). b) Plots of the relative fluorescence intensity (*I*/*I*_0_) of *pR*‐TPE‐P5, *pS*‐TPE‐P5, *pR*‐polymer and *pS*‐polymer versus *f*_w_. Inset: the associated fluorescence photographs in solution and aggregate states (*f*_w_ = 90%) taken under 365 nm UV irradiation. c) The fluorescence quantum yields (*Φ*, histogram) and fluorescence lifetime data (scatter plots) of *pR*‐TPE‐P5, *pS*‐TPE‐P5, *pR*‐polymer, and *pS*‐polymer in solution and aggregate states. d) CD spectra of *pR*‐TPE‐P5, *pS*‐TPE‐P5 and their polymers in solution and aggregate states (*f*_w_ = 90%). e) CPL spectra of *pR*‐TPE‐P5 and *pS*‐TPE‐P5 in solution and aggregate states (*f*_w_ = 90%). f) CPL spectra of *pR*‐TPE‐P5, *pS*‐TPE‐P5 and their polymers in solution and aggregate states (*f*_w_ = 90%). g) CPL and h) *g*_lum_ spectra of *pR*‐polymer in aqueous solutions with different *f*_w_. i) *|g*_lum_ *|* values of the solution/aggregates of *pR*‐TPE‐P5, *pS*‐TPE‐P5, *pR*‐polymer, and *pS*‐polymer. The scattered data in all CPL and *g*_lum_ spectra represents the raw data and the solid lines are the nonlinear fitting curves obtained by the Gaussian algorithm. Solution concentration: 10 µM.

Inspired by the above results, we further examined the effect of aggregation behavior on the luminescent and chiroptical properties of the AIEgen‐functionalized pillar[5]arene monomers and polymers. The UV‐vis absorption spectra of the polymers showed little change after aggregation (Figure S25). The PL spectra of *pR*‐TPE‐P5, *pS*‐TPE‐P5, *pR*/*pS*‐TPE‐P5, and their coordination polymers were then measured and compared in DMSO and DMSO/water mixtures with different water fractions (*f*_w_). As depicted in Figure 5a‒5b and Figures S26‒S29, the PL intensity of all these materials was significantly boosted with the increase of water content, showing typical AIE behaviors. The formation of aggregates in aqueous media with a water content of 90% was further confirmed by the dynamic light scattering (DLS) measurements, and average particle sizes of *p*R/*p*S‐TPE‐P5 and *p*R/*p*S‐polymer aggregates were measured to be 142 and 396 nm, respectively (Figure S30). At high water fractions, the PL intensity of polymers slightly decreased possibly due to the extensive formation of polymer aggregates under such conditions, which led to a decline in the effective solute concentration.^[^ ^50^ ^]^ No obvious change in the peak pattern was observed after aggregation. The aggregates of both monomers and polymers exhibited bright bluish green fluorescence at ∼500 nm with obviously higher *Φ* values and lifetime than those of their solutions. As summarized in Table S2 and **Figure** **5c**, the *Φ* values of the monomers and polymers can reach about 43% and 49%, respectively, in the aggregate state, and the fluorescence lifetime greatly increased from 0.26‒0.35 ns to 2.92‒3.71 ns after aggregation (Figure S31). These results suggested that the increased local concentration and rigidity of AIEgens in pillar[5]arenes could impede the non‐radiative energy transfer processes, thus facilitating the improvement of PL performance.^[^ ^51^, ^52^ ^]^

The CD spectra shown in **Figure** **5d** indicated that the occurrence of physical aggregation slightly decreased the CD signals of the AIEgen‐functionalized pillar[5]arene enantiomers and their polymers, possibly due to the change in their morphologies at different conditions. As depicted in Figure S32, in solution state, the self‐assembly morphologies of *pR*‐ or *pS*‐polymer obviously change from helical fibers into a mixture dominated by spherical morphology. The macroscopic isotropy of spheres is unfavorable for the chiral property of the assemblies, thus leading to a slight decrease of the CD signals. By contrast, a pair of mirror CPL signals emerged at ∼510 nm with |*g*_lum_| values in the range of 1.25 × 10^−3^ after the aggregation of *pR*‐ and *pS*‐TPE‐P5 in aqueous media (**Figure** **5e** and Figure S33), showing an aggregation‐induced CPL phenomenon. A similarly aggregation‐enhanced CPL effect was also observed in the CPL results of *pR*‐ and *pS*‐polymers. As depicted in Figure S34, the CPL intensity of the coordination polymers increased about 8 folds upon aggregation accompanied with a 21‐fold increase in |*g*_lum_| value to be 2.69 × 10^−3^. The thin films of *pR*‐polymer and *pS*‐polymer also showed good CPL performance with *g*_lum_ values of 3.72 × 10^−3^ and −3.30 × 10^−3^ at 510 nm, respectively (Figure S35). A collective comparison between the CPL spectra of monomers with those of polymers in different states (**Figure** **5f**) clearly demonstrated the synergistic contribution of polymerization and aggregation for the boosting of CPL. In order to investigate the detailed variation tendency of the CPL performance of polymers with different degrees of aggregation, we further measured the CD and CPL spectra at aqueous solutions with different *f*_w_ by taking *pR*‐polymer as an example. The CD signal slightly decreased with the increase of *f*_w_ (Figure S36), whereas the CPL intensity and *g*_lum_ value of *pR*‐polymer steadily increased as the increased of *f*_w_ from 0% to 80%, reaching the maximum of *g*_lum_ (3.91 × 10^−3^ at 510 nm) at *f*_w_ = 80%. Further increasing *f*_w_ to 90% led to the decrease of CPL intensity and *g*_lum_ to a certain extent (**Figure** **5g**‐**5h**). In sum, the polymerization process or the physical aggregation process of the AIEgen‐functionalized pillar[5]arenes can increase their *Φ* and |*g*_lum_| values individually. By simultaneously utilizing the positive effects of polymerization and aggregation on the PL and CPL performance of this system, pillar[5]arene‐based chiral supramolecular polymers with high *Φ* of up to 49% and high |*g*_lum_| of 2.69 × 10^−3^ in aggregate states can be readily achieved (Figure 5c and **Figure** **5i**). Based on the recently proposed concept of CPL brightness (*B*_CPL_), the overall performance of the present CPL polymer emitters at aggregate states could be evaluated using the equation of *B*_CPL_ = *ε*_abs_ × *Φ* × |*g*_lum_|/2, ^[^ ^53^, ^54^ ^]^ where *ε*_abs_ denotes the molar extinction coefficient of the sample at the maximum absorption wavelength measured with an integrating sphere (Figure S24). With all these necessary parameters in hand, the aggregate‐state *B*_CPL_ of the chiral supramolecular polymers was calculated to be ∼11.2 M^−1^ cm^−1^, indicating their great potential to serve as excellent emitter for CPL applications.^[^ ^53^ ^]^

**Figure 6:**
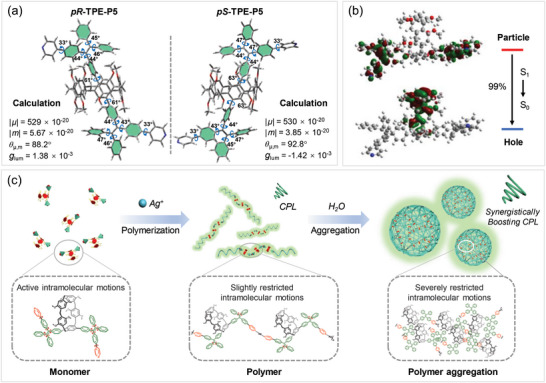
a) The optimized S_1_ geometries of *pR*‐TPE‐P5 and *pS*‐TPE‐P5 labelled with different torsion angles and their calculated transition dipole moments for the S_1_ → S_0_ transition. The unit for the electric transition dipole moment (*µ*) and magnetic transition dipole moment (*m*) is esu cm and erg G^−1^, respectively. b) Natural transition orbitals of *pR*‐TPE‐P5 calculated with the optimized S_1_ geometry by TD‐DFT at the wB97XD/def2‐SVP level. The percentage refers to the proportion of the dominant particle‐to‐hole transition. c) Schematic diagram of the possible mechanism of polymerization‐ and aggregation‐enhanced CPL.

To gain insight into the underlying structure‐property relationship of the polymerization/aggregation‐enhanced CPL, theoretical models were constructed and different CPL‐related parameters were calculated by time‐dependent density functional theory (TD‐DFT) using Gaussian 16 program package. As the polymer structure is too complex, the pair of enantiomer monomers (*pR*‐ and *pS*‐TPE‐P5) were selected as the theoretical models for discussion. Firstly, the optimal configurations of *pR*‐ and *pS*‐TPE‐P5 at singlet excited states with minimum energy points were calculated at B97XD/def2‐SVP level. As depicted in **Figure** **6a**, the pyridyl‐TPE substituent bilaterally aligned in the pillar[5]arene unit adopts a highly twisted conformation with multiple rotors. The torsion angles between the pyridyl‐TPE substituents and pillar[5]arene were measured to be 61°‒63° in the optimized configurations, which indicates that these twisted and bulky substituents are able to spatially limit the oxygen‐through‐the‐annulus rotations of the hydroquinone rings of pillar[5]arene. At the optimized S_1_ geometries of the enantiomer monomers, three CPL‐relevant parameters, including the electric transition dipole moment (*µ*), the magnetic transition dipole moment (*m*), and the vector angle of *µ* and *m* (i.e., *θ*_µ,m_) were then calculated (Figure 6a and Figure S37). The calculated |*µ*| and |*m*| for *pR*‐TPE‐P5 is 529 × 10^−20^ esu cm and 5.67 × 10^−20^ erg G^−1^, respectively, while the calculated *θ*_µ,m_ is 88.2°, in line with its positive CPL signal. According to the simplified formula: *g*_lum_ = 4|*m*|cos*θ_µ,m_*/|*µ*|,^[^ ^55^, ^56^ ^]^ the *g*_lum_ of *pR*‐TPE‐P5 at its optimized S_1_ geometry was calculated to be 1.38 × 10^−3^, and the *g*_lum_ value of *pS*‐TPE‐P5 was predicated to be −1.42 × 10^−3^. These calculation results are in good consistency with the experimental results of *pR*‐TPE‐P5 and *pS*‐TPE‐P5 in constrained states such as after polymerization or aggregation (Figure S23 and Figure S33). The theoretical models of TPE‐P5 enantiomers disregard the intramolecular motions by using fixed S_1_ geometries, thus rendering them artificially limited to show simulation results of the quasi‐constrained states. Furthermore, the geometrical properties and electronic properties of singlet excited states of the silver‐coordinated dimer of *pR*‐TPE‐P5 were calculated to provide some hints for the CPL change tendency of properties of the assembled structure. As depicted in Figure S39, Compared with the CPL‐relevant parameters of *pR*‐TPE‐P5 monomer, the formation of dimer has little influence on the |*µ*| value but obviously changes the |m| value and the vector angle (*θ*_µ,m_). The calculated |*µ*| was reduced by 3.8 times after dimerization, and the calculated *θ*_µ,m_ of *pR*‐TPE‐P5 dimer was 0.7° smaller than that of *pR*‐TPE‐P5. The corresponding *g*_lum_ value of *pR*‐TPE‐P5 dimer at its optimized S_1_ geometry was determined to be 2.44×10^−3^, which was 1.8 times higher than that of the *pR*‐TPE‐P5 monomer. These dimer‐based calculation results further suggested that the formation of assemblies or polymers was beneficial to enhance the *g*_lum_ values. Meanwhile, the analysis in the natural transition orbitals of monomers from S_1_ to S_0_ were calculated at the same level of theory (**Figure** **6b** and Figure S38). Taking *pR*‐TPE‐P5 as an example, during the S_1_ → S_0_ transition, the “particle” is mainly distributed in the pyridyl‐TPE moiety (electron donor), while the “hole” is concentrated on the skeleton of pillar[5]arene (electron acceptor). The large separation between “hole” and “particle” implied that the TPE‐P5 enantiomers exhibited strong donor‐acceptor charge transfer transition, making their electronic properties susceptible to the change of local environment, thereby affecting the CPL performance.^[^ ^57^ ^]^ The intramolecular motions of the multiple rotors in the electron donor (AIEgen) moiety would remarkably influence the *g*_lum_ values. Based on the abovementioned experimental and theoretical results, we proposed a possible mechanism for the synergistic effect of polymerization‐ and aggregation‐enhanced CPL. As illustrated in **Figure** **6c**, the negligible CPL signals of enantiomerically pure TPE‐P5 in dilute solutions may be ascribed to the active intramolecular motions of AIEgen and other moieties at such conditions, which is unfavorable for both the fluorescence and chiral properties of the functionalized pillar[5]arenes. The formation of coordination polymers restricts the intramolecular motions to some extent due to the larger steric hindrance and the presence of multiple intra‐/intermolecular interactions of polymers, which contributes to the better chirality fixation of pillar[5]arenes as well as higher fluorescence efficiency to show obvious CPL properties. Additional aggregation of supramolecular polymers further rigidifies the molecular conformations and strengthens the RIM effect due to the physical constraint, eventually leading to a significant boosting of the CPL performance.

## 3 Conclusion

In summary, a novel supramolecular coordination polymer system with efficient CPL in aggregate states was developed based on the planar chirality and excellent fluorescence properties of the AIEgen‐modified pillar[5]arenes. The introduction of pyridyl‐TPE unit into pillar[5]arene not only endows the corresponding monomers and polymers with AIE properties, but also restricts the active oxygen‐through‐the‐annulus rotations of the hydroquinone rings of pillar[5]arene by steric effects, thus providing convenience for obtaining the planar chirality of pillar[5]arene monomers (*pR*‐TPE‐P5 and *pS*‐TPE‐P5) by chiral HPLC. Further coordination reaction between the pyridyl‐TPE‐pillar[5]arene monomers and silver ions affords supramolecular polymers with obviously enhanced CD signals and fluorescence intensity than their monomers. The CD signals in solution state increase by 1.5 folds and the *Φ* values in solution state increase from ∼0.9 to ∼2.0 after polymerization. The dual enhancement in chiral and fluorescence properties induces the remarkable CPL signals of *pR*‐polymer and *pS*‐polymer with |*g*_lum_| values of 1.30 × 10^−4^ and −0.65 × 10^−4^, respectively, at 510 nm in dilute solutions. Upon the formation of aggregates in aqueous media, the *Φ* values of the polymer further increase from ∼1.9% to ∼49%, while the |*g*_lum_| values significantly increased by ∼21 folds to be 2.69 × 10^−3^ at 510 nm for *pR*‐polymer. The theoretical simulation results revealed that the CPL properties of this system are susceptible to the change of local environment. The RIM effects of the multiple rotors in the AIEgen moiety and the pillar[5]arene unit together with the aggregation‐induced conformation confinement collectively contribute to the boosting of *g*_lum_ values by polymerization and aggregation. Such a synergistic effect of polymerization‐ and aggregation‐enhanced CPL provides a simple and feasible strategy to achieve high‐performance CPL‐active supramolecular polymer emitters, paving the pathway for further research on the design and construction of diversified macrocyclic polymers with highly efficient aggregate‐state CPL properties and multiple functionalities.

## Conflict of Interest

The authors declare no conflict of interest.

## Supporting information



Supporting Information

## Data Availability

The data that support the findings of this study are available from the corresponding author upon reasonable request.
