# Supplementary material for: Synergistically Boosting the Circularly Polarized Luminescence of Functionalized Pillar[5]arenes by Polymerization and Aggregation
Source: Adv Sci (Weinh). 2023 Oct 22;10(35):2305149. doi: 10.1002/advs.202305149 (PMC10724438; doi:10.1002/advs.202305149)
Supplement: Supplementary file 1 — Supporting Information [file ADVS-10-2305149-s001.pdf]

ADVANCED SCIENCE
Open Access

Supporting Information

for *Adv. Sci.*, DOI 10.1002/advs.202305149

Synergistically Boosting the Circularly Polarized Luminescence of Functionalized Pillar[5]arenes by Polymerization and Aggregation

Hewei Yan, Xiaojun Yin, Dong Wang, Ting Han and Ben Zhong Tang**

Synergistically Boosting the Circularly Polarized Luminescence of Functionalized Pillar[5]arenes by Polymerization and Aggregation

Hewei Yan, [a] [b] Xiaojun Yin,[a] Dong Wang,[a] Ting Han* [a] and Ben Zhong Tang* [c]

[a] Center for AIE Research, Shenzhen Key Laboratory of Polymer Science and Technology, Guangdong Research Center for Interfacial Engineering of Functional Materials, College of Materials Science and Engineering, Shenzhen University, Shenzhen, Guangdong 518060, China.
[b] College of Physics and Optoelectronic Engineering, Shenzhen University, Shenzhen 518060, China
[c] School of Science and Engineering, Shenzhen Institute of Aggregate Science and Technology, The Chinese University of Hong Kong, Shenzhen (CUHK-Shenzhen), Guangdong 518172, China

E-mail: hanting@szu.edu.cn; tangbenz@cuhk.edu.cn

Table of Contents

1. Experimental Section 3

1.1 Materials 3

1.2 Instruments 3

2. Synthesis and Structural Characterization 4

2.1 Synthesis and characterization of *pR*/*pS*-TPE-P5 4

2.2 Preparation of chiral *pR*-TPE-P5 and *pS*-TPE-P5 11

2.3 Synthesis and characterization of supramolecular polymers 12

3. Theoretical Calculations of the Ground States 18

4. Photophysical Properties 18

5. Theoretical Calculations of the Singlet Excited States 27

Reference..29

1. Experimental Section

1.1 Materials

Paraformaldehyde, boron (tri)fluoride etherate, 1,2-dichloroethane, dichloromethane, potassium carbonate, tetrahydrofuran, and thionyl chloride were purchased from China National Medicines Corporation Ltd. Trifluoromethanesulfonic anhydride, 1,1-diphenyl-2,2-di(p-bromophenyl)ethylene, pyridine-4-boronic, bis(triphenylphosphine)palladium(II) chloride, 1,1'-bis(diphenylphosphino)ferrocene]dichloropalladium(II), and tetrakis (triphenylphosphine)palladium were purchased from Bide Pharmatech Ltd. Sodium hydrosulfite and deuterated chloroform were purchased from Macklin Inc. Pyridine, bis(pinacolato)diboron, and silver nitrate were purchased from Aladdin Ltd. 1,4-dimethoxybenzene was purchased from Meryer Ltd.

1.2 Instruments

^{1}H NMR and ^{13}C NMR spectra were recorded on Bruker AVANCE III 600 MHz, AVANCE III HD 500 MHZ and AVANCE NEO 400MHZ at 298 K using $CDCl_3$ as the solvent. Chemical shifts (δ/ppm) were calibrated using the residual solvent peak as the internal standard ($CDCl_3$: δ = 7.26 ppm for ^{1}H NMR spectra and 77.16 ppm for ^{13}C NMR spectra). Electrospray ionization mass spectrometry (ESI-MS) was performed by a DFS high resolution FD-MS (Thermo Fisher Scientific, Bremen, Germany) operating in the positive ion mode. MALDI-TOF-MS results were recorded on a Bruker autoflex speed. The resolution of *p*R/*pS*-TPE-P5 was performed by the Daicel Chiral Technologies (China) Co., Ltd. Circular dichroism (CD) spectra were measured on Bio-Logic MOS-450. Fourier transform infrared (FTIR) spectra were obtained on a Nicolet 6700 spectrometer in the range of 4000-525 cm^{-1} over 128 scans. X-ray photoelectron spectroscopy (XPS) spectra were recorded on a VG Scientific X-ray photoelectron spectrometer (Model ESCALab220i-XL). UV-visible absorption spectra were recorded on a SHIMADZU UV-2600i UV-Vis spectrophotometer. The photoluminescence (PL) spectra and lifetime data were measured on an Edinburgh Instruments FLS1000 spectrophotometer. Fluorescence quantum yields were determined using a Hamamatsu C11347 spectrometer. Circularly polarized luminescence spectra were obtained on JASCO CPL300. The scanning electron microscope（SEM）images were recorded on a Thermo APREO S microscope, and the sample used for SEM imaging was prepared by dripping the solution (1 μM) onto a silicon wafer followed by natural evaporation for 24 h and then plating with gold via Leica EM ACE600.

2. Synthesis and Structural Characterization

2.1 Synthesis and characterization of pR/pS-TPE-P5

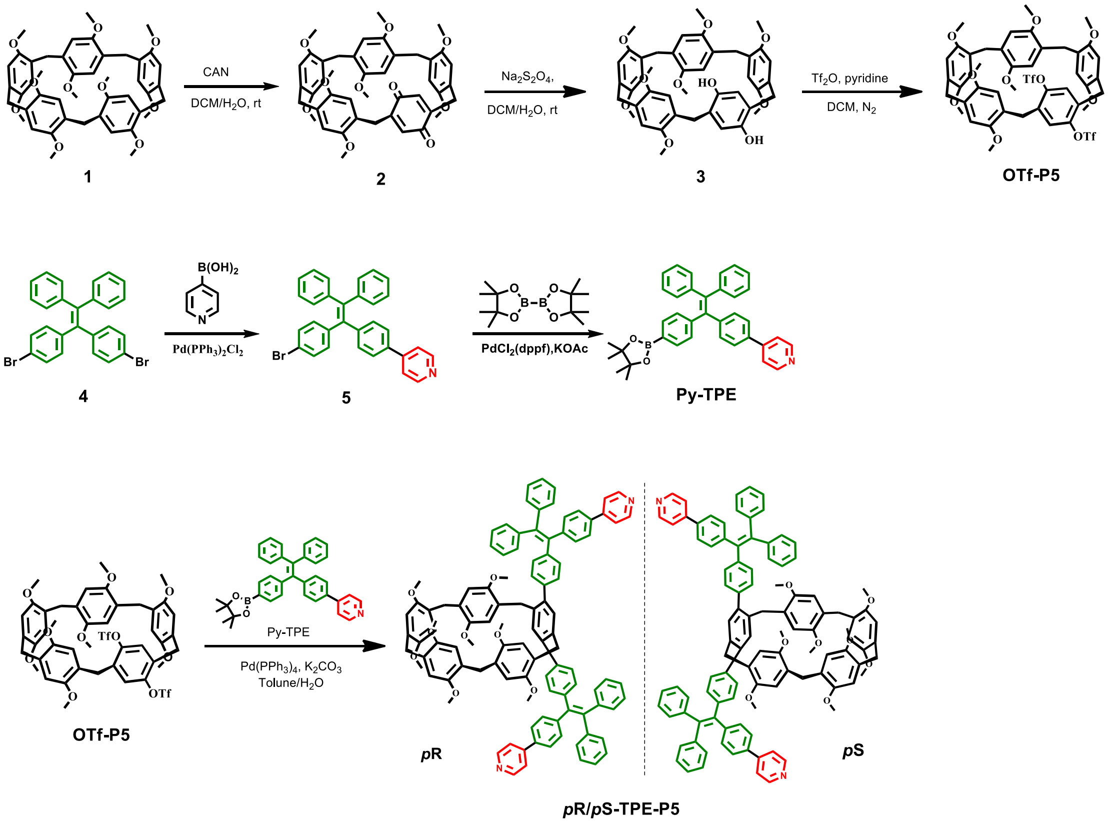

Figure S1. Synthetic routes toward compound *pR/pS*-TPE-P5.

Synthesis and characterization of compound 2: Compound **1** was synthesized according to the reported procedures in literature.[1] Compound **1** (1 g, 1.33 mmol) was first added to 60 mL dichloromethane (DCM) in a 150 mL flask, into which the aqueous solution of ammonium ceric nitrate (2.92 g, 5.33 mmol) was then added dropwise at room temperature under stirring. After reacting for 1 h, 100 mL H_2O was added to the mixture and the resulting mixture was extracted with DCM for 3 times. The organic phase was collected and then dried over anhydrous Na_2SO_4. After removing the solvent by rotary evaporation and further purification by the silica gel column chromatography (eluent: petroleum ether (PE)/ethyl acetate (EA) = 5/1, v/v), compound **2** (750 mg, yield = 78%) was finally obtained as red powder. ^{1}H NMR (400 MHz, $CDCl_3$, 298 K), δ (ppm): 6.84 (s, 2H), 6.81 (s, 2H), 6.79 (s, 2H), 6.67 (d, J = 4.4 Hz, 4H), 3.79 (d, J = 3.2 Hz, 7H), 3.75 (s, 6H), 3.71 (d, J = 0.8 Hz, 13H), 3.63 (s, 6H), 3.59 (s, 4H).

Synthesis and characterization of OTf-P5: OTf-P5 was synthesized according to the reported procedures in literature[1]. Compound **2** (0.75 g, 1.04 mmol) was dissolved in 15 mL DCM in a 50 mL flask. Under N_2 atmosphere, then the aqueous solution of $Na_2S_2O_4$ (1.81 g, 10.4 mmol) was added into the above mixture dropwise under stirring and under

N_2 atmosphere. When the mixture changed from red to colorless, the reaction was stopped by adding 100 mL H_2O to the mixture. The mixture was then extracted with DCM for 3 times. The organic phase was collected and then dried over anhydrous Na_2SO_4. After removing the solvent by rotary evaporation, the crude product was processed to the next step without further purification. The crude product and pyridine (1 mL) were added to 15 mL anhydrous DCM, and then trifluoromethanesulfonic anhydride (2 mL) was added dropwise to the mixture at 0°C. Next, the reaction was allowed to stir for 12 h at room temperature. The reaction was quenched by adding water followed by extracting the mixture with DCM for 3 times. After drying the mixture over anhydrous Na_2SO_4, the solvent of the organic phase was removed by rotary evaporation. OTf-P5 was eventually obtained as white powder (550 mg, yield = 54%) after further purification by the silica gel column chromatography (eluent: PE/EA = 10/1, v/v). ^1H NMR (600 MHz, $CDCl_3$, 298 K), δ (ppm): 7.35 (s, 2H), 6.82 (s, 2H), 6.80 (s, 2H), 6.78 (s, 2H), 6.71 (s, 2H), 3.86 (s, 4H), 3.80 (d, J = 4.2 Hz, 6H), 3.74 (s, 6H), 3.70 (s, 6H), 3.68 (s, 6H), 3.63 (s, 6H). ^{13}C NMR (150 MHz, $CDCl_3$, 298 K), δ (ppm): 150.88, 150.84, 150.82, 150.61, 146.30, 133.95, 129.78, 128.47, 128.01, 124.66, 124.24, 114.16, 113.95, 113.76, 113.70, 55.88, 55.81, 55.79, 55.24, 30.69, 29.64, 29.38. MALDI-TOF HRMS (m/z): calcd. for $C_{105}H_{88}N_2O_3$ $[M]^+$ 1505.6574, found 1505.6570.

Synthesis and characterization of compound 5: Compound **4** (2 g, 4.08 mmol), K_2CO_3 (1.41 g, 10.20 mmol), $Pd(PPh_3)_2Cl_2$ (143 mg, 204 μmol) and pyridine-4-boronic (476 mg, 3.88 mmol) were sequentially added into a 100 mL flask equipped with a magnetic stir bar under N_2 atmosphere. Then the solvent mixture of THF/H_2O (v/v = 4:1, 50 mL) was added into the reaction system. Next, the temperature was raised to 80 °C and the mixture was reacted for 72 h. Afterward, the reaction was cooled to room temperature and quenched by adding water into the mixture. After being extracted with DCM following by drying over anhydrous Na_2SO_4, the organic phase was collected and the solvent was removed by rotary evaporation. With further purification by the silica gel column chromatography (eluent: PE/EA = 10/1, v/v), compound **5** (1.1 g, yield = 55%) was obtained as white powder. ^1H NMR (600 MHz, $CDCl_3$, 298 K), δ (ppm): 8.62 (d, J = 4.8 Hz, 2H), 7.47 (d, J = 5.4 z, 2H), 7.41 (d, J = 8.4 z, 2H), 7.25–7.23 (m, 2H), 7.18–7.09 (m, 8H), 7.06–7.01 (m, 4H), 6.92 (d, J = 8.4 Hz, 2H). ^{13}C NMR (150 MHz, $CDCl_3$, 298 K), δ (ppm): 149.98, 148.13, 144.60, 143.29, 143.20, 142.63, 142.50, 138.86, 135.91, 133.13, 132,24, 131.35, 131.16, 128.07, 128.02, 127.05, 127.03, 126.45, 121.51, 120.85.

Synthesis and characterization of Py-TPE: Compound **5** (1.1 g, 2.25 mmol), CH_3COOK (1.33 g, 13.51 mmol), Pd(dppf)Cl_2 (165 mg, 225 μmol) and bis(pinacolato)diboron (1.72 g, 6.76 mmol) were sequentially added into 30 mL anhydrous DMSO in a 100 mL flask equipped with a magnetic stir bar under N_2 atmosphere. After raising the temperature to 100 °C, the mixture was allowed to react for 24 h. Then the reaction was cooled to room temperature and quenched by adding water into the mixture. After the mixture was extracted with DCM and dried over anhydrous Na_2SO_4, the organic phase was collected and the solvent was removed by rotary evaporation. Py-TPE (1.05 g, yield = 87%) was finally obtained as yellow soild by further purification using the silica gel column

chromatography (eluent: PE/EA = 10/1, v/v). ^{1}H NMR (500 MHz, $CDCl_3$, 298 K), δ (ppm): 8.61 (d, J = 4.0 Hz, 2H), 7.57 (d, J = 8.0 Hz, 2H), 7.48 (d, J = 6.0 Hz, 2H), 7.39 (d, J = 8.5 Hz, 2H), 7.14–7.09 (m, 8H), 7.08–7.03 (m, 6H), 1.32 (s, 12H). ^{13}C NMR (125 MHz, $CDCl_3$, 298 K), δ (ppm): 149.82, 148.36, 146.55, 143.56, 143.39, 142.42, 140.04, 135.63, 134.37, 132.30, 131.46, 131.41, 130.86, 127.98, 127.92, 126.89, 126.32, 121.56, 83.90, 25.04.

Synthesis of pR/pS-TPE-P5: OTf-P5 (250 mg, 253 μmol), Py-TPE (271 mg, 506 μmol), $Pd(PPh_3)_4$ (58.5 mg, 51 μmol), and K_2CO_3 (106 mg, 760 μmol) were sequentially added into a 10 mL flask under N_2 atmosphere. Then 5 mL solvent mixture of THF/H_2O (v/v = 4:1) was added into the reaction system. Next, the reaction temperature was stirred for 24 h at 80°C. After cooling to room temperature, the reaction was quenched by adding water and then extracted with DCM for 3 times. The organic layer was dried over anhydrous Na_2SO_4 and subjected to a rotary evaporator to remove the solvent. After further purification by the silica gel column chromatography (eluent: DCM/MeOH = 10/1, v/v), *pR/pS-TPE-P5* (105 mg, yield = 31%) was obtained as yellow solid. ^{1}H NMR (600 MHz, $CDCl_3$, 298 K), δ (ppm): 8.64 (d, J = 3.6 Hz, 4H), 7.50 (d, J = 6.0 Hz, 4H), 7.47 (d, J = 7.8 Hz, 4H), 7.23 (d, J = 8.4 Hz, 4H), 7.19 (s, 2H), 7.17–7.08 (m, 20 H), 7.06 (d, J = 7.8 Hz, 4H), 6.96 (d, J = 7.8 Hz, 4H), 6.71 (s, 2H), 6.67 (s, 2H), 6.61 (s, 2H), 5.94 (s, 2H), 3.85–3.70 (m, 10H), 3.57 (s, 6H), 3.51 (s, 6H), 3.47 (s, 6H), 3.29 (s, 6H). ^{13}C NMR (150 MHz, $CDCl_3$, 298 K), δ (ppm): 171.26, 151.21, 151.05, 150.97, 150.77, 150.14, 147.97, 145.05, 143.60, 143.57, 142.17, 141.77, 140.02, 139.84, 136.44, 135.80, 132.29, 131.45, 131.42, 131.07, 129.16, 128.80, 128.20, 127.98, 127.86, 126.34, 121.44, 114.47, 114.33, 114.27, 114.20, 56.24, 56.13, 55.99, 55.74, 32.28, 30.59, 29.82, 29.58, 21.18. ESI-HRMS (*m*/*z*): calcd. for $C_{105}H_{88}N_2O_3$ $[M]^+$ 1505.6574, found 1505.6570.

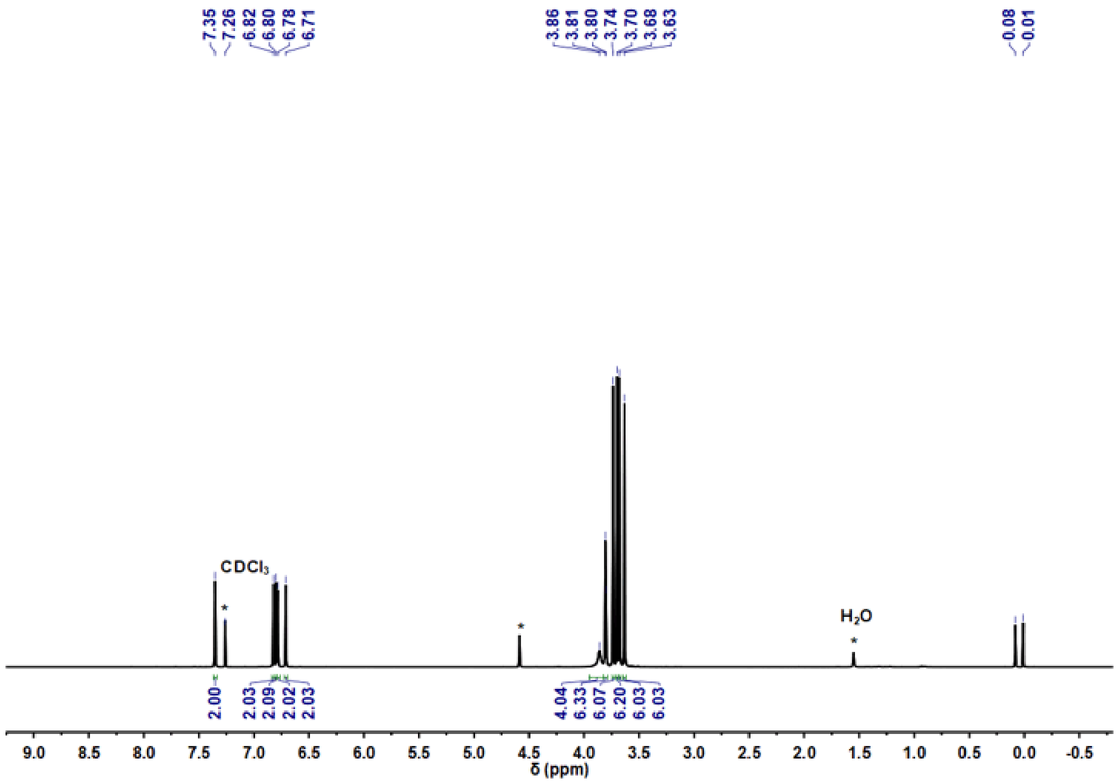

Figure S2. The ^{1}H NMR spectrum of OTf-P5 ($CDCl_3$, 600 MHz, 298 K). The solvent peaks are marked with asterisks.

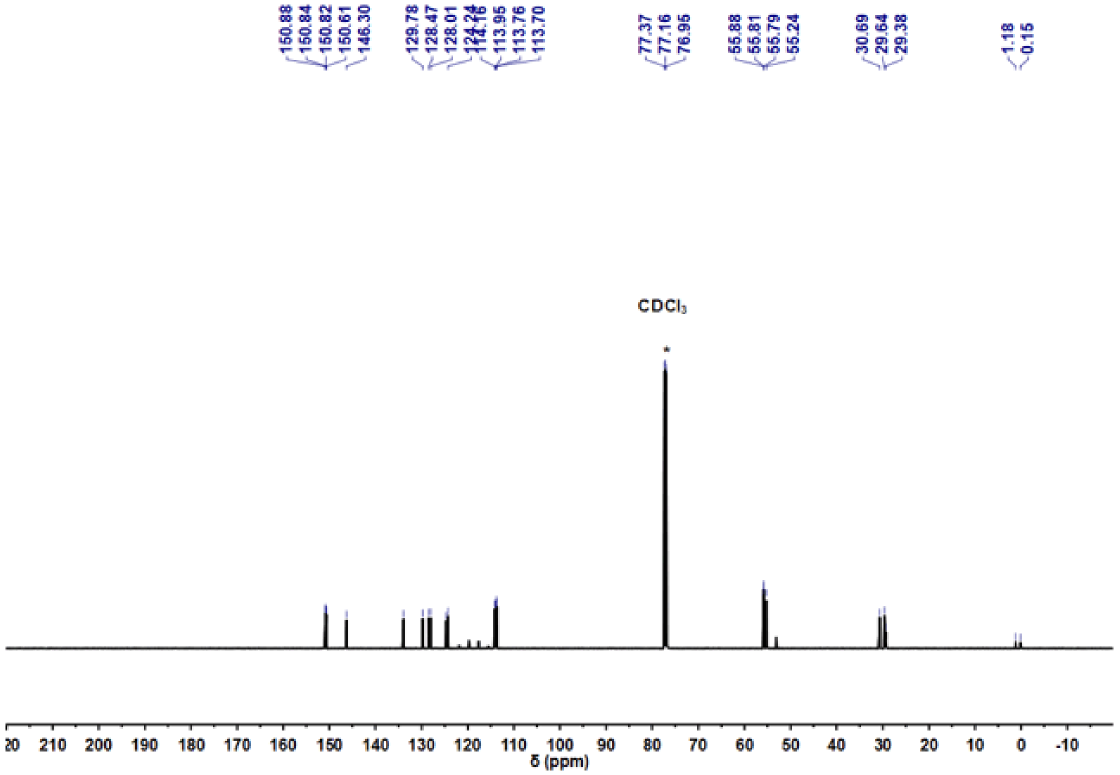

Figure S3. The ^{13}C NMR spectrum of OTf-P5 ($CDCl_3$, 150 MHz, 298 K). The solvent peaks are marked with asterisks.

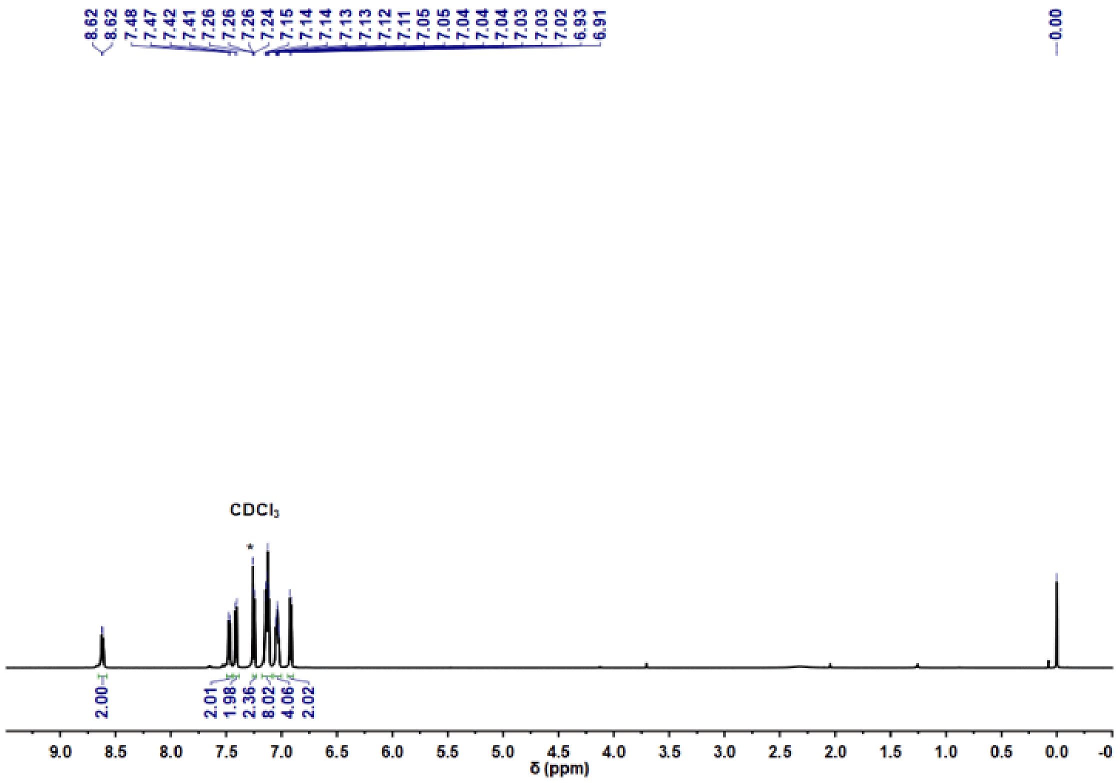

Figure S4. The ^{1}H NMR spectum of compound **5** ($CDCl_3$, 600 MHz, 298 K). The solvent peaks are marked with asterisks.

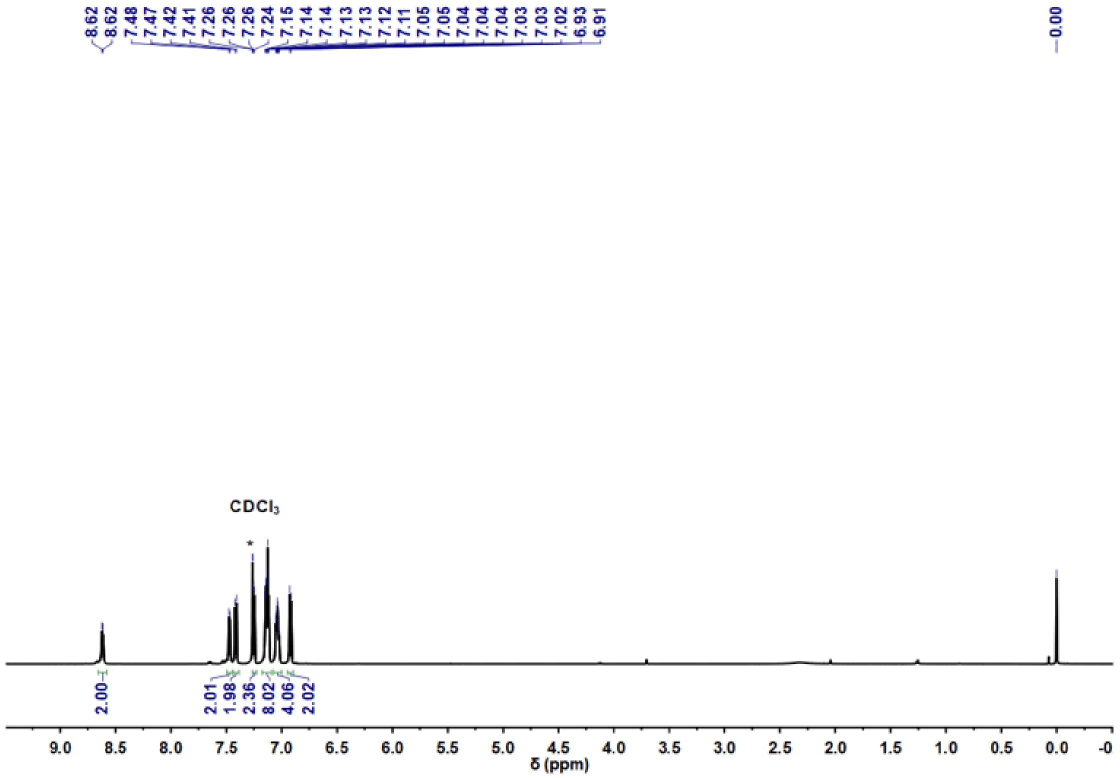

Figure S5. The ^{13}C NMR spectrum of compound **5** ($CDCl_3$, 150 MHz, 298 K). The solvent peaks are marked with asterisks.

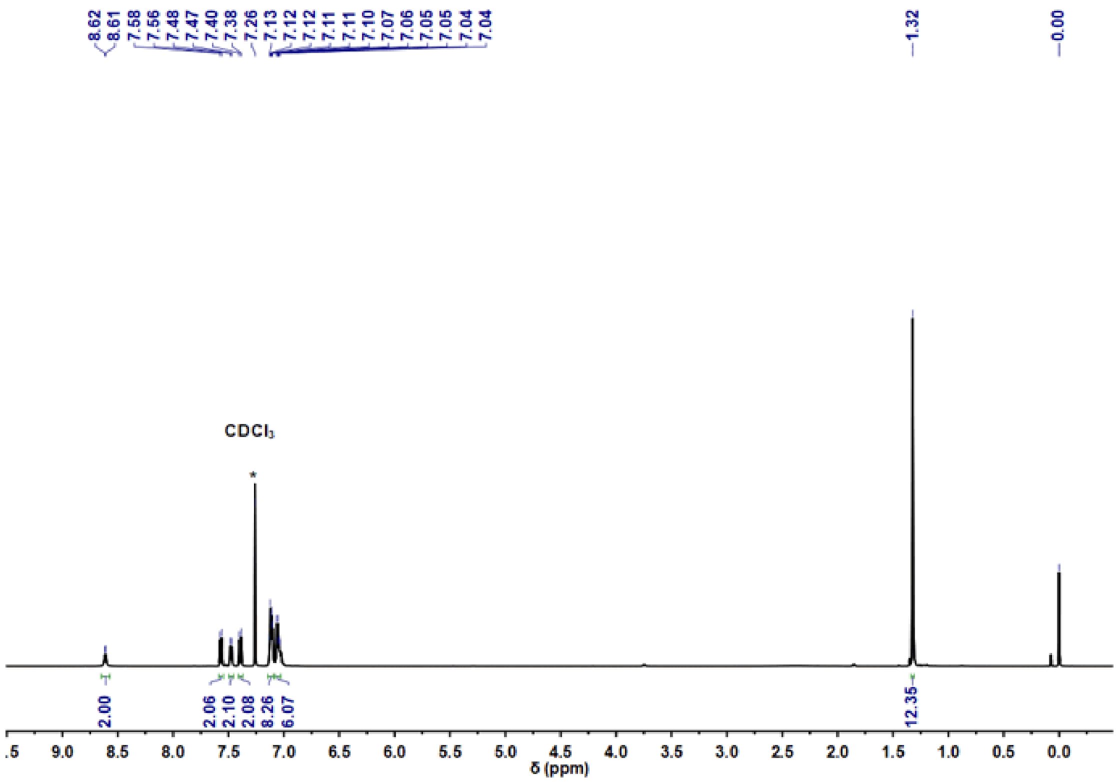

Figure S6. The ^{1}H NMR spectrum of Py-TPE ($CDCl_3$, 500 MHz, 298 K). The solvent peaks are marked with asterisks.

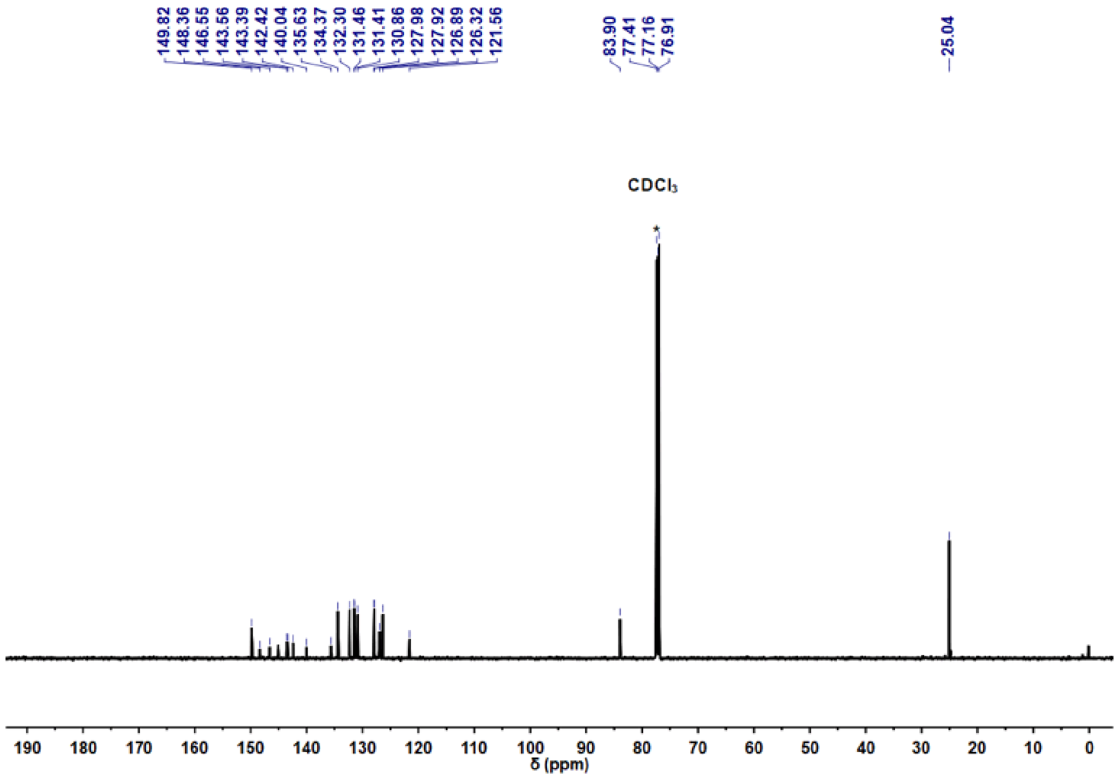

Figure S7. The ^{13}C NMR spectrum of Py-TPE ($CDCl_3$, 125 MHz, 298 K). The solvent peaks are marked with asterisks.

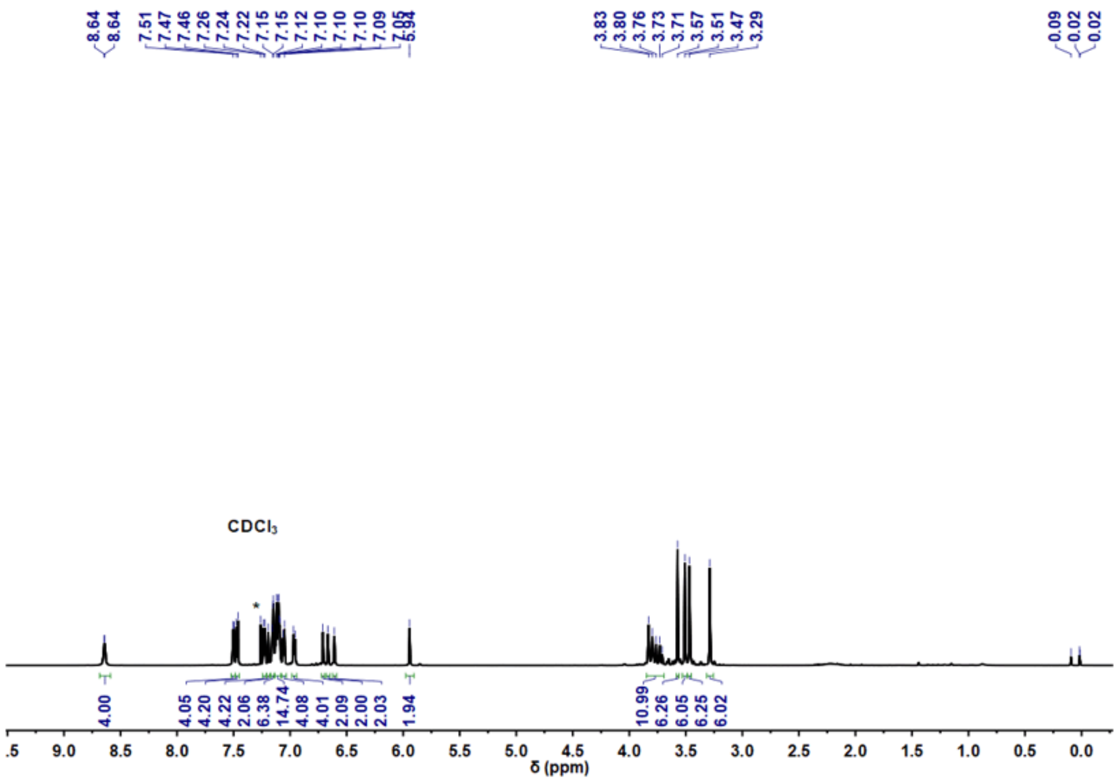

Figure S8. The ^{1}H NMR spectrum of *pR*/*pS*-TPE-P5 ($CDCl_3$, 600 MHz, 298 K). The solvent peaks are marked with asterisks.

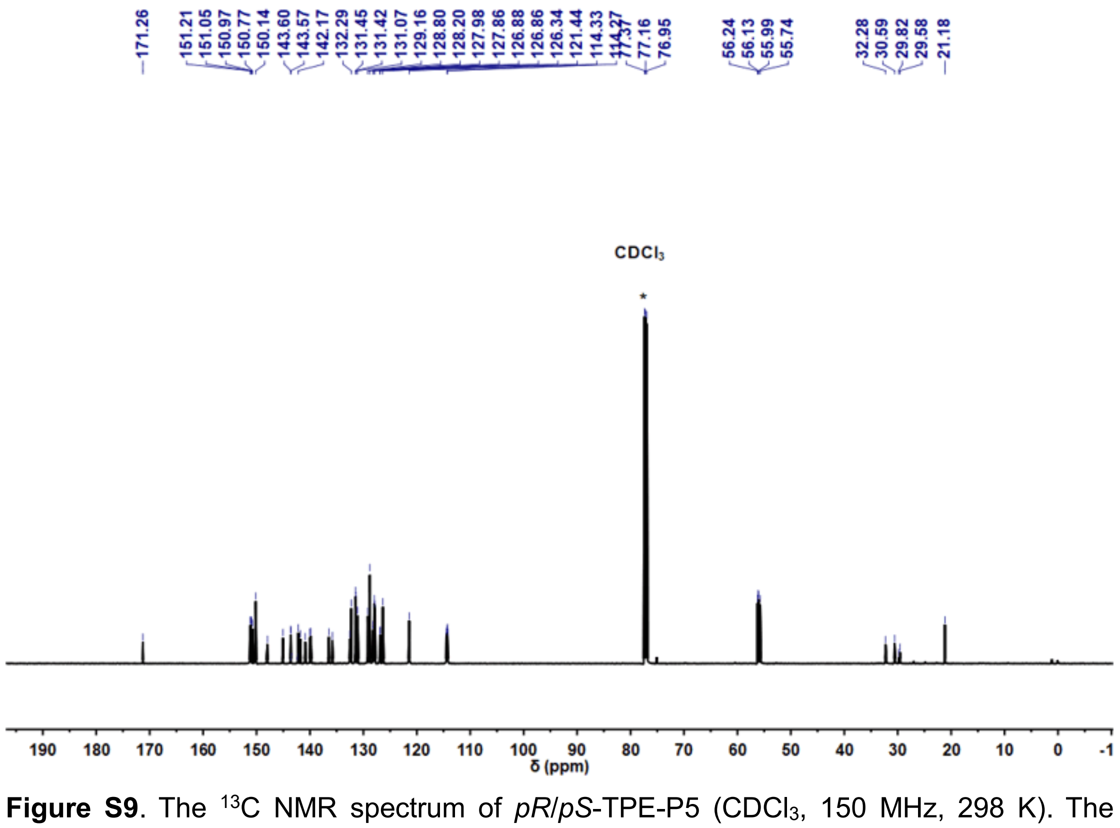

Figure S9. The ^{13}C NMR spectrum of *pR*/*pS*-TPE-P5 ($CDCl_3$, 150 MHz, 298 K). The solvent peaks are marked with asterisks.

2.2 Preparation of chiral pR-TPE-P5 and pS-TPE-P5

The resolution of *pR*/*pS*-TPE-P5 was performed by Daicel Chiral Technologies (China) Co., Ltd. on a chiral HPLC equipped with a CHIRALPAK IG preparation column using DCM:MeOH = 1:1 as the eluent.[2] Flow rate: 1.0 mL/min. Temperature: 25°C. The chromatograms before resolving are shown below.

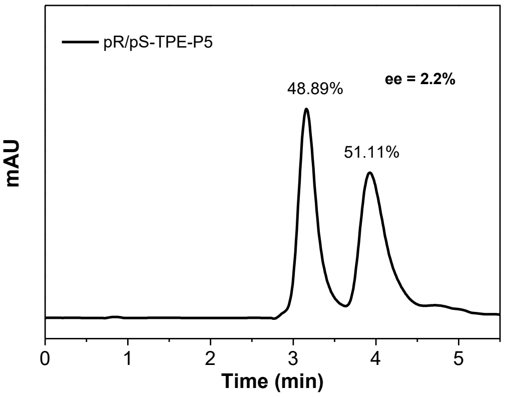

Figure S10. High performance liquid chromatography data of *pR*/*pS*-TPE-P5.

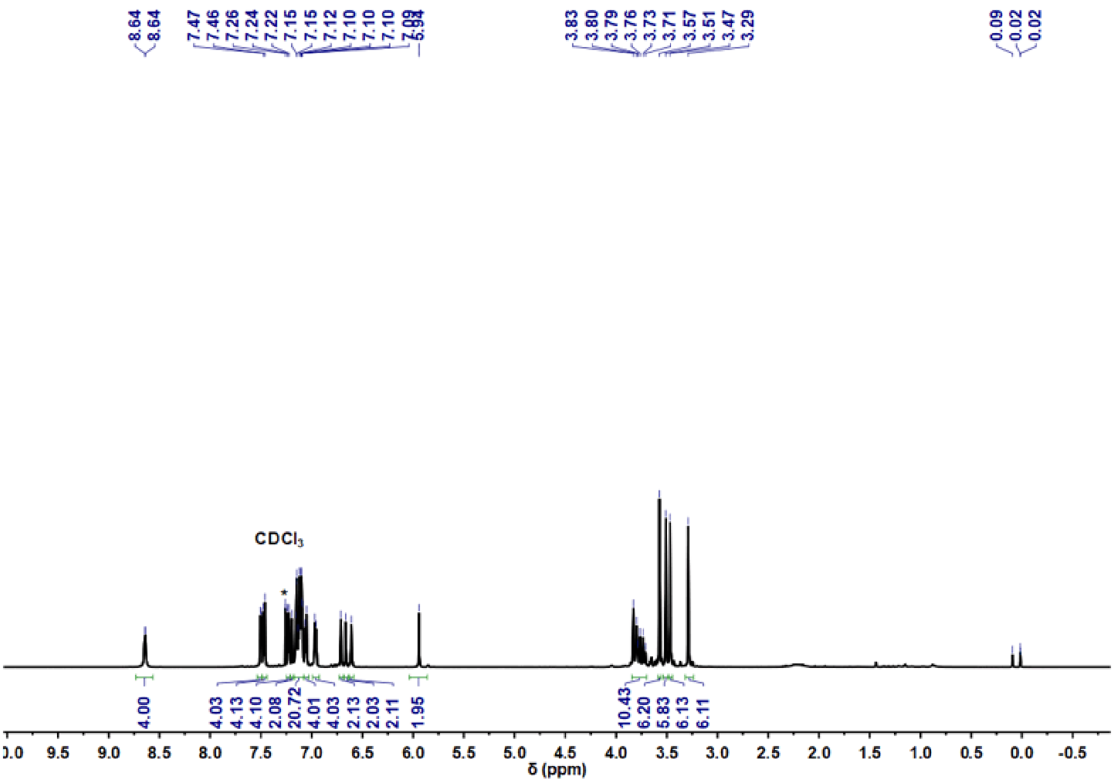

Figure S11 The ^{1}H NMR spectrum of *pR*-TPE-P5 ($CDCl_3$, 400 MHz, 298 K). The solvent peaks are marked with asterisks.

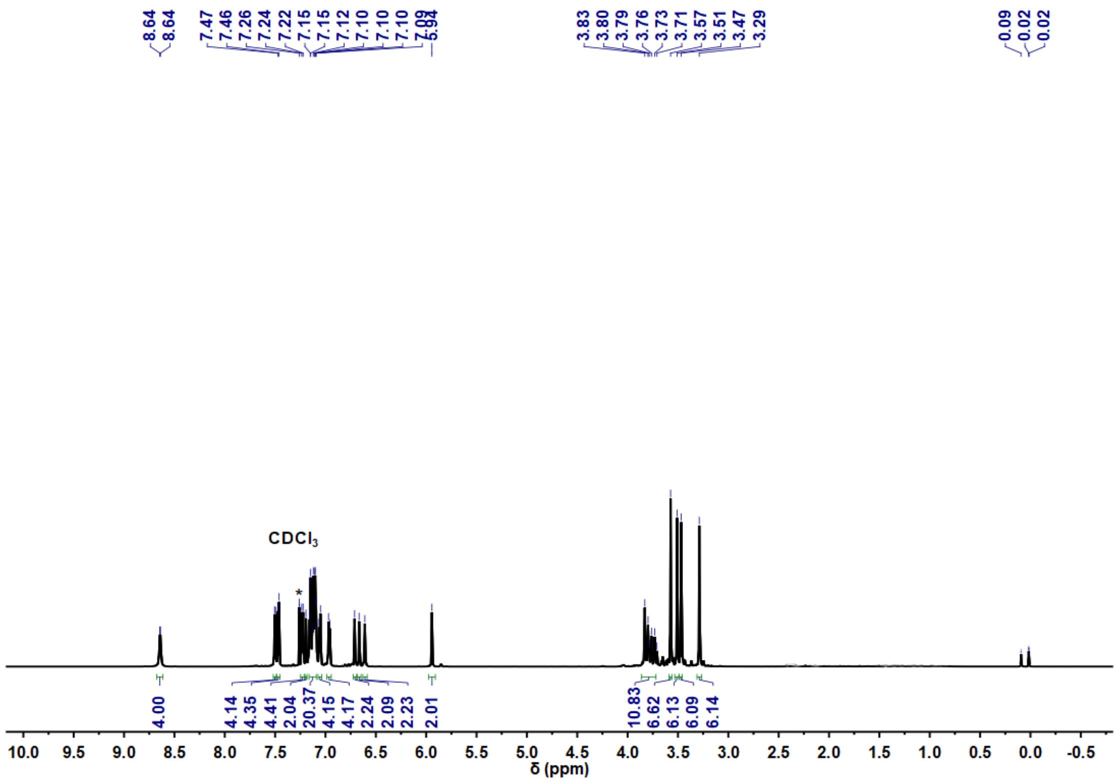

Figure S12 The ^{1}H NMR spectrum of *pS*-TPE-P5 ($CDCl_3$, 400 MHz, 298 K). The solvent peaks are marked with asterisks.

2.3 Synthesis and characterization of supramolecular polymers

Synthesis and characterization of pR/pS-polymer: *pR*/*pS*-TPE-P5 (10 mg, 6.64 μM) and silver nitrate (112 mg, 664 μM) were added to a single-neck flask with a volume of 50 mL. Then 10 mL of a mixed solution of THF/H_2O (v/v, 1:1) was added, and the mixture is stirred thoroughly at room temperature for 24 hours. After removing the solvent using a rotary evaporator, an appropriate amount of dichloromethane was added to flask. The mixture was washed with distilled water to remove excess inorganic salts. The organic phases were combined and anhydrous sodium sulfate was added for drying. After solvent removal, vacuum drying was performed, resulting in 8.0 mg of yellow solid *pR*/*pS*-polymer (yeild 75%). ^{1}H NMR (400 MHz, $CDCl_3$, 298 K), *δ* (ppm): 8.66 (s, 4H), 7.67 (s, 4H), 7.49 (d, *J* = 6.8 Hz, 4H), 7.24 (s, 2H), 7.18–7.06 (m, 24H), 7.02 (d, *J* = 6.4 Hz, 4H), 6.94 (d, *J* = 6.8 Hz, 4H), 6.67 (s, 2H), 6.62 (s, 2H), 6.55 (s, 2H), 5.94 (s, 2H), 3.85–3.71 (m, 10H), 3.54 (s, 6H), 3.47 (s, 6H), 3.42 (s, 6H), 3.26 (s, 6H).

Synthesis and characterization of pR-polymer: *pR*-TPE-P5 (10 mg, 6.64 μM) and silver nitrate (112 mg, 664 μM) were added to a single-neck flask with a volume of 50 mL. Then 10 mL of a mixed solution of THF/H_2O (v/v = 1:1) was added, and the mixture is stirred thoroughly at room temperature for 24 hours. After removing the solvent using a rotary evaporator, an appropriate amount of dichloromethane was added to flask. The mixture was washed with distilled water to remove excess inorganic salts. The organic phases were combined and anhydrous sodium sulfate was added for drying. After solvent removal,

vacuum drying was performed, resulting in 7.5 mg of yellow solid *pR*-polymer (yeild 70%). ^{1}H NMR (400 MHz, $CDCl_3$, 298 K), δ (ppm): 8.66 (s, 4H), 7.67 (s, 4H), 7.49 (d, J = 6.8 Hz, 4H), 7.24 (s, 2H), 7.17 – 7.07 (m, 24H), 7.02 (d, J = 6.4 Hz, 4H), 6.94 (d, J = 6.8 Hz, 4H), 6.66 (s, 2H), 6.62 (s, 2H), 6.54 (s, 2H), 5.94 (s, 2H), 3.84 – 3.70 (m, 10H), 3.54 (s, 6H), 3.47 (s, 6H), 3.42 (s, 6H), 3.26 (s, 6H).

Synthesis and characterization of pS-polymer: *pS*-TPE-P5 (10 mg, 6.64 μM) and silver nitrate (112 mg, 664 μM) were added to a single-neck flask with a volume of 50 mL. Then 10 mL of a mixed solution of THF/H_2O (1:1) was added, and the mixture is stirred thoroughly at room temperature for 24 hours. After removing the solvent using a rotary evaporator, an appropriate amount of dichloromethane was added to flask. The mixture was washed with distilled water to remove excess inorganic salts. The organic phases were combined and anhydrous sodium sulfate was added for drying. After solvent removal, vacuum drying was performed, resulting in 7.7 mg of yellow solid *pS*-polymer (yeild 72%). ^{1}H NMR (400 MHz, $CDCl_3$, 298 K), δ (ppm): 8.66 (s, 4H), 7.67 (s, 4H), 7.49 (d, J = 6.8 Hz, 4H), 7.24 (s, 2H), 7.16 – 7.06 (m, 24H), 7.02 (d, J = 6.4 Hz, 4H), 6.94 (d, J = 6.8 Hz, 4H), 6.66 (s, 2H), 6.62 (s, 2H), 6.54 (s, 2H), 5.94 (s, 2H), 3.85–3.71 (m, 10H), 3.54 (s, 6H), 3.47 (s, 6H), 3.42 (s, 6H), 3.26 (s, 6H).

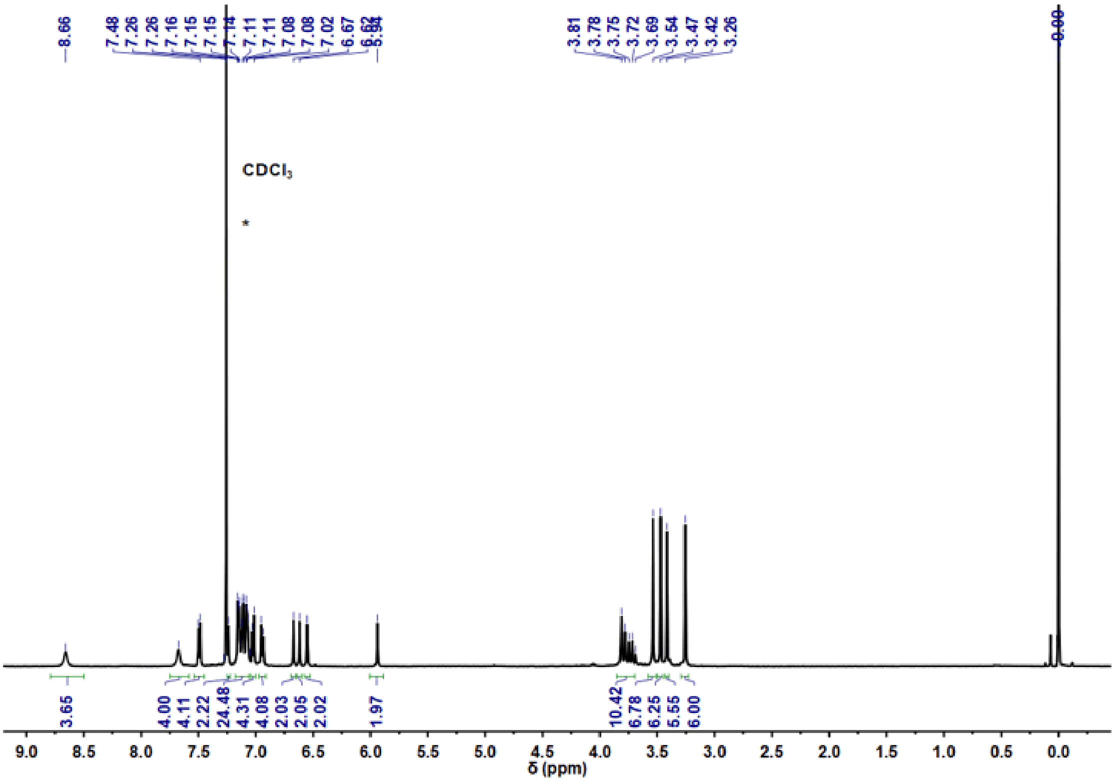

Figure S13 The ^{1}H NMR spectrum of *pR/pS*-polymer ($CDCl_3$, 400 MHz, 298 K). The solvent peaks are marked with asterisks.

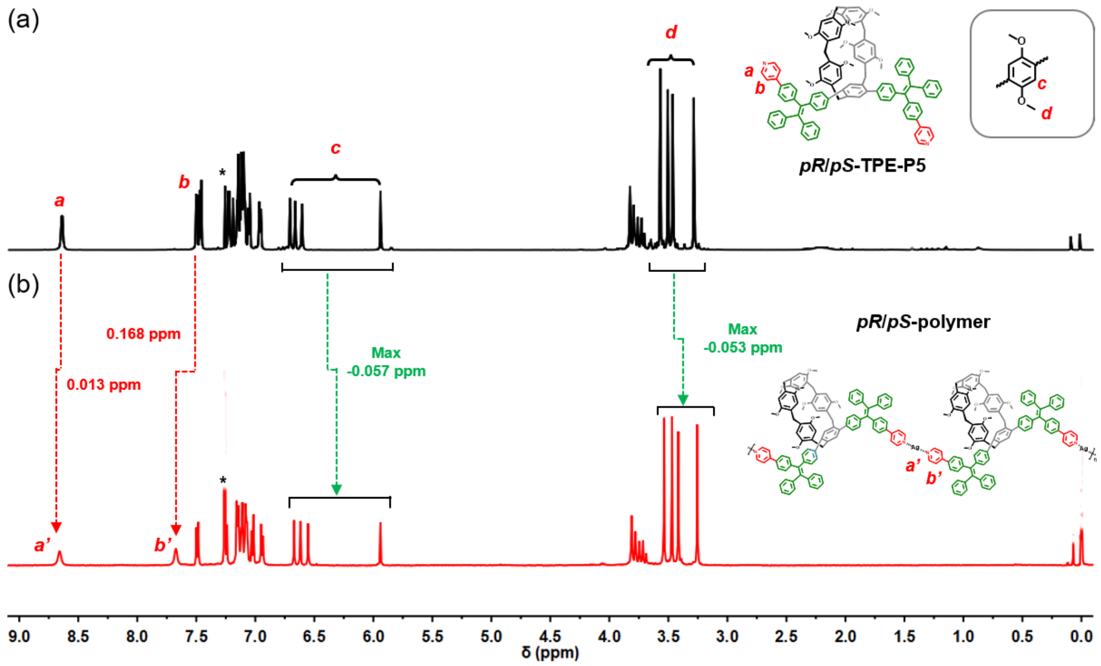

Figure S14. ^{1}H NMR spectra of (a) *pR*/*pS*-TPE-P5 and (b) *pR*/*pS*-polymer ($CDCl_3$, 400 MHz, 298 K). The solvent peaks are marked with asterisks.

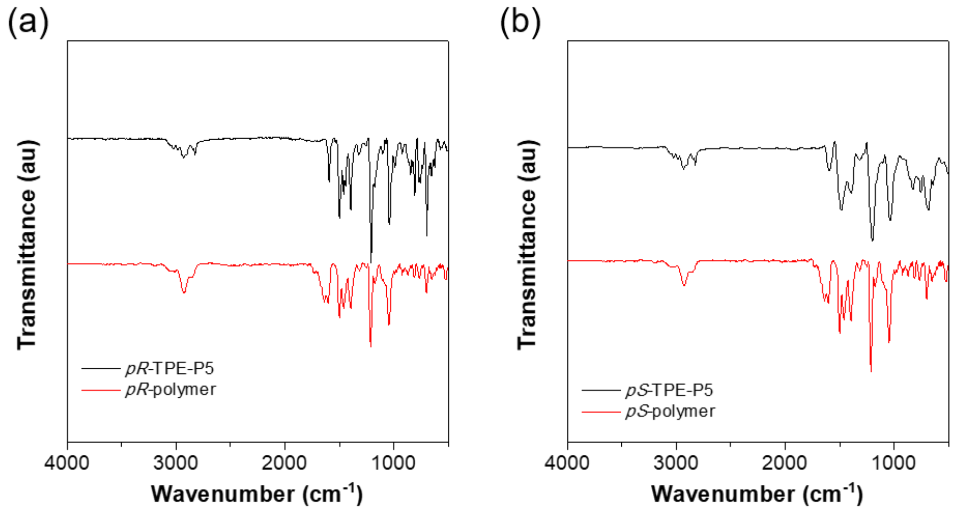

Figure S15. (a) FT-IR spectra of *pR*-TPE-P5 and *pR*-polymer. (b) FT-IR spectra of *pS*-TPE-P5 and *pS*-polymer.

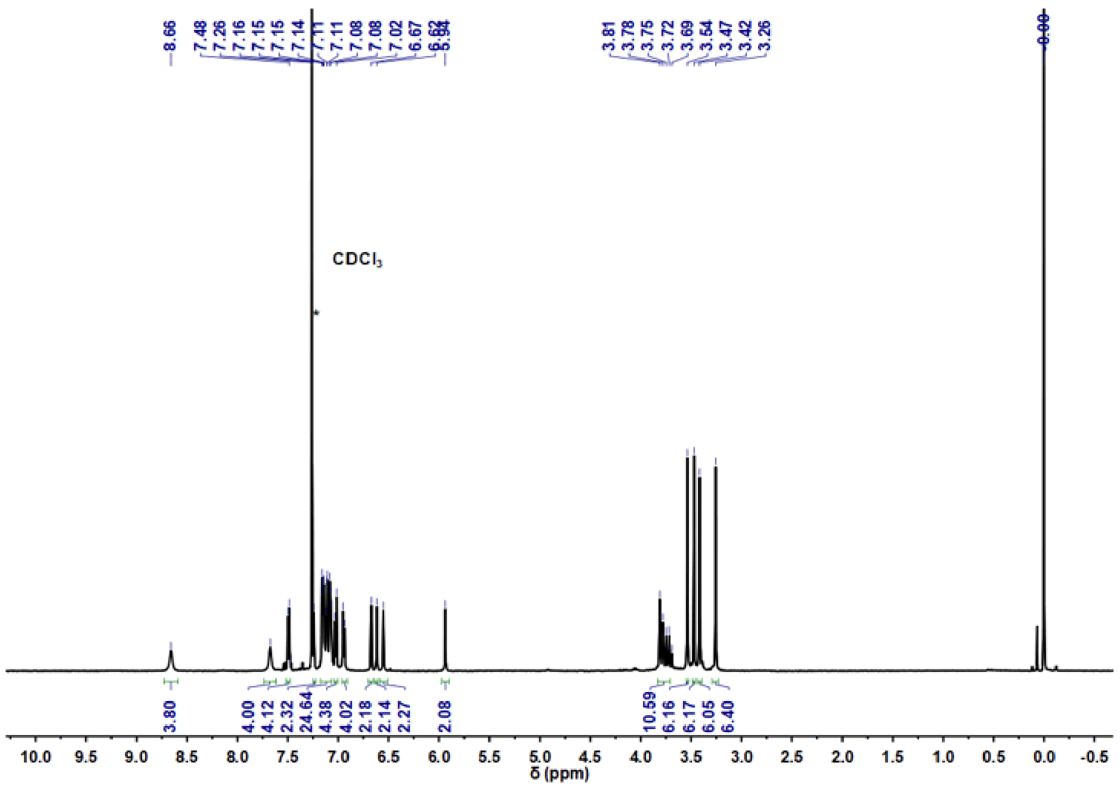

Figure S16. The ^{1}H NMR spectrum of *pR*-polymer ($CDCl_3$, 400 MHz, 298 K). The solvent peaks are marked with asterisks.

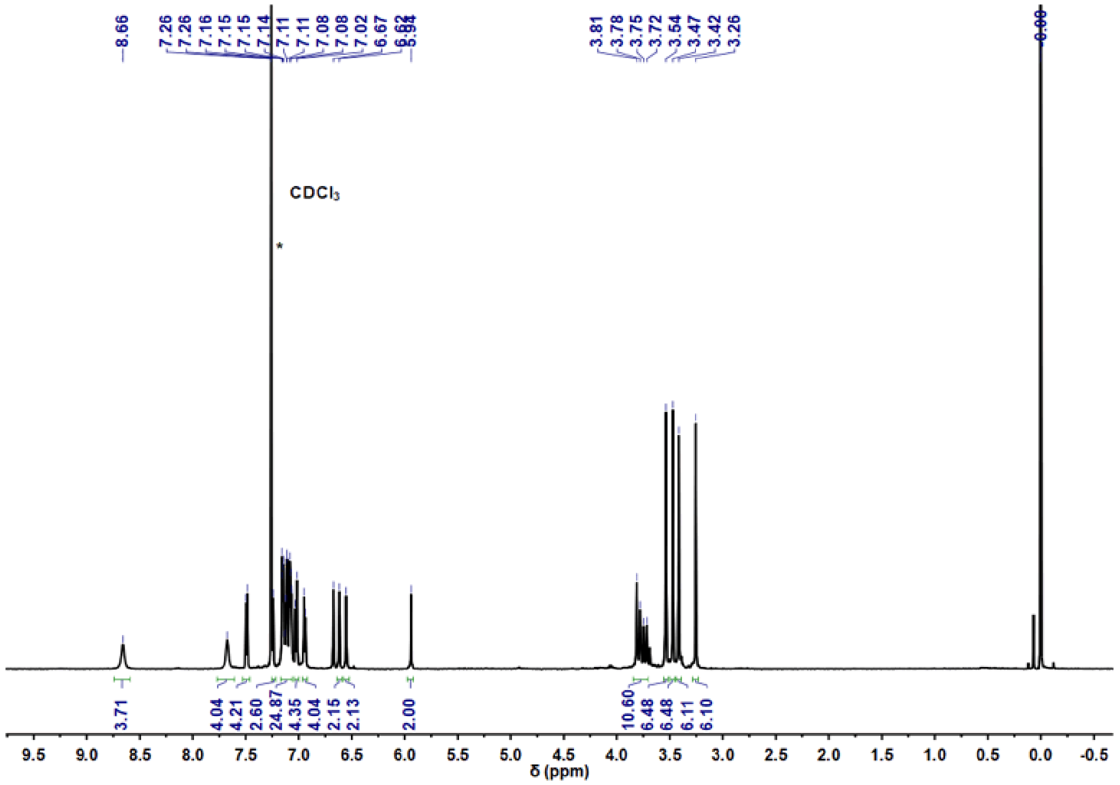

Figure S17. The ^{1}H NMR spectrum of *pS*-polymer ($CDCl_3$, XX MHz, 298 K). The solvent peaks are marked with asterisks.

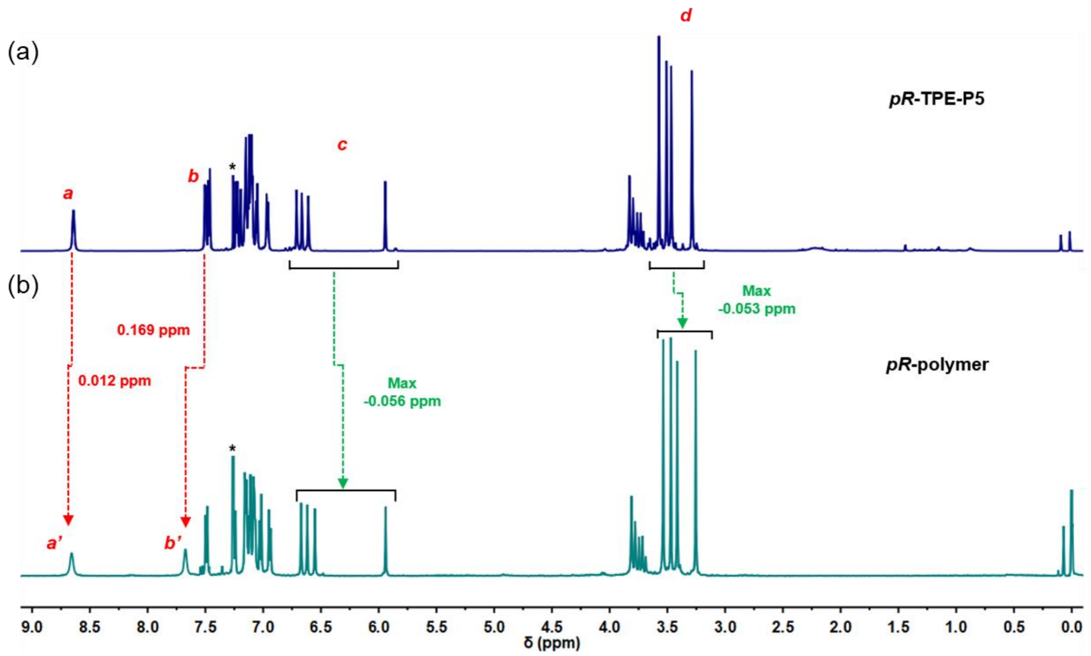

Figure S18. ^{1}H NMR spectra of (a) *pR*-TPE-P5 and (b) *pR*-polymer ($CDCl_3$, 400 MHz, 298 K). The solvent peaks are marked with asterisks.

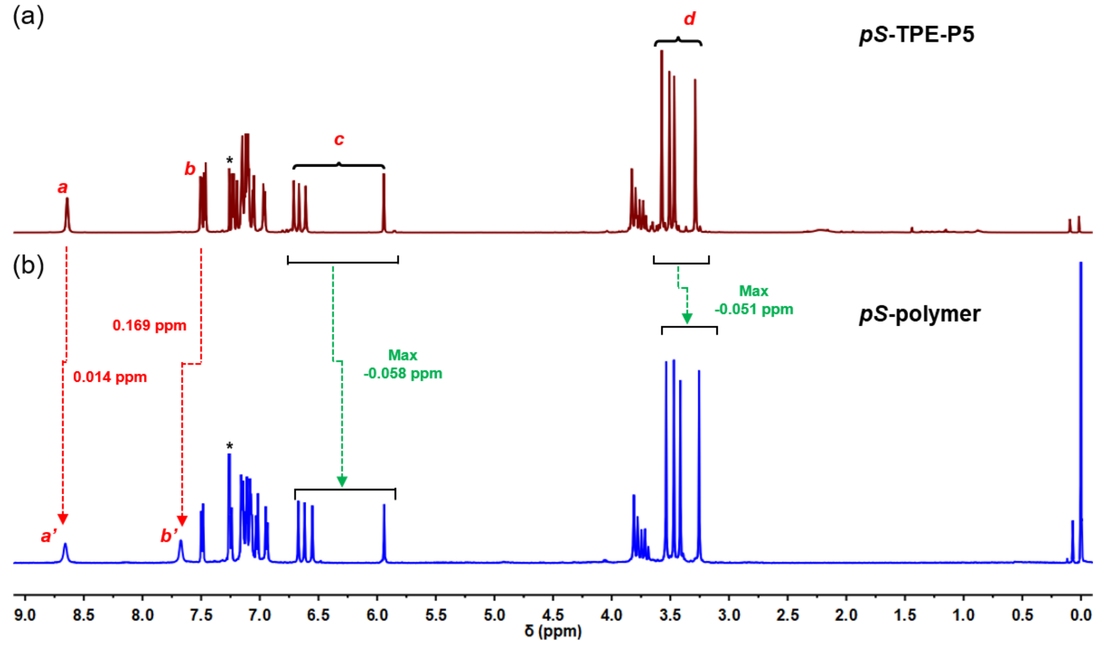

Figure S19. ^{1}H NMR spectra of (a) *pS*-TPE-P5 and (b) *pS*-polymer ($CDCl_3$, 400 MHz, 298 K). The solvent peaks are marked with asterisks.

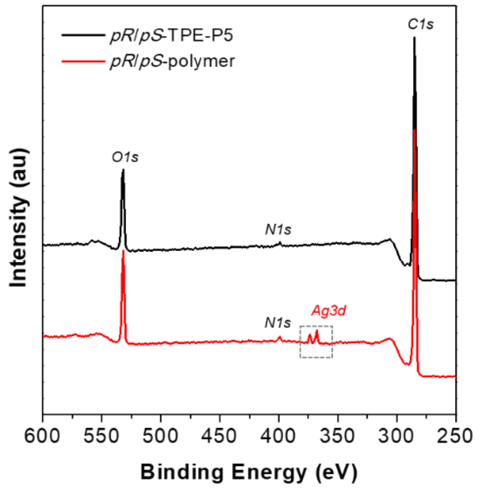

Figure S20 XPS spectra of *pR*/*pS*-TPE-P5 and *pR*/*pS*-polymer.

3. Theoretical Calculations of the Ground States

Density functional theory (DFT) calculations of *pR*- and *pS*-TPE-P5 were carried out by using Gaussian 16 program package.[3] The DFT calculations on the geometrical properties of the ground state were performed based on B3LYP density functional method including Grimme's dispersion correction with def2-SVP basis set. The UV-vis absorption and CD spectra were simulated with the optimized S_0 geometries by the time-dependent DFT (TD-DFT) calculations at the wB97XD/def2-SVP level of theory.

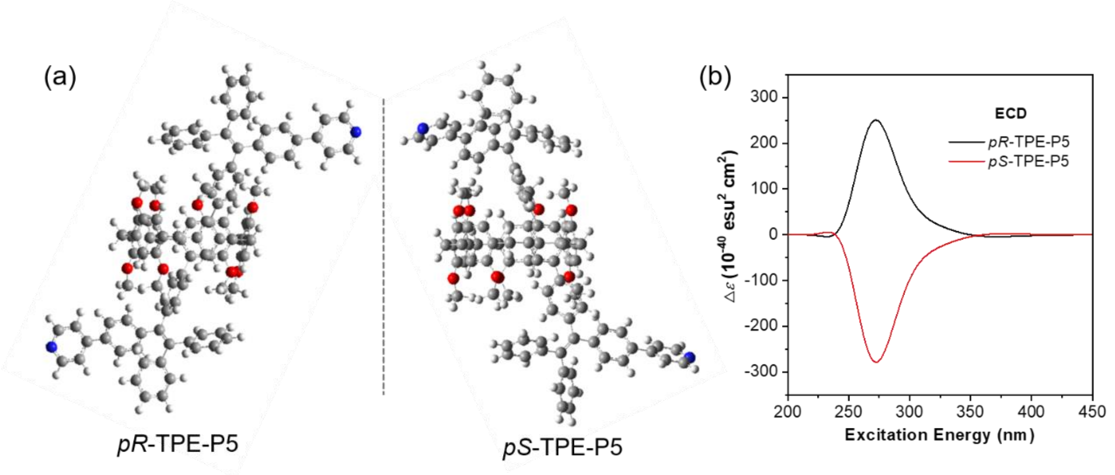

Figure S21. (a) The optimized ground state (S_0) geometries of *pR*-TPE-P5 and *pS*-TPE-P5. (b) The simulated ECD absorption spectra of *pR*- and *pS*-TPE-P5 by TD-DFT at the wB97XD/def2-SVP level.

4. Photophysical Properties

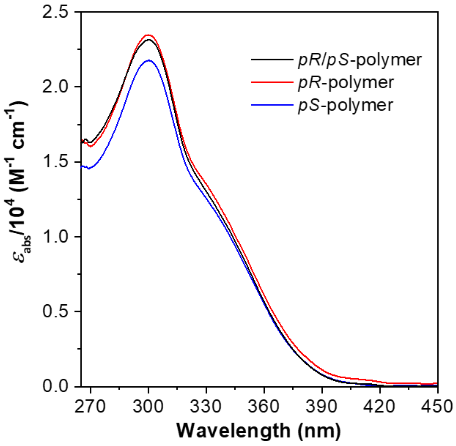

Figure S22 The UV-vis absorption spectra of *pR*-polymer, *pS*-polymer and *pR*/*pS*-polymer in DMSO solution. Concentration: 10 μM.

Table S1 Fluorescence quantum yields (Φ) of the monomers and polymers.

Parameter	TPE-P5 in solution[a]			Polymer in solution		
	pR	*pS*	*pR/pS*	*pR*	*pS*	*pR/pS*
Φ (%)	0.9	1.0	0.9	1.9	1.8	2.0
**Parameter**	**TPE-P5 aggregates[b]**			**Polymer aggregates**		
	pR	*pS*	*pR/pS*	*pR*	*pS*	*pR/pS*
Φ (%)	43.2	43.6	43.3	49.1	49.4	49.3

[a] Solvent: DMSO. Concentration: 10 μM. Excitation wavelength = 298 nm.

[b] Solvent: H_2O/DMSO = 90/10. Concentration: 10 μM. Excitation wavelength = 298 nm.

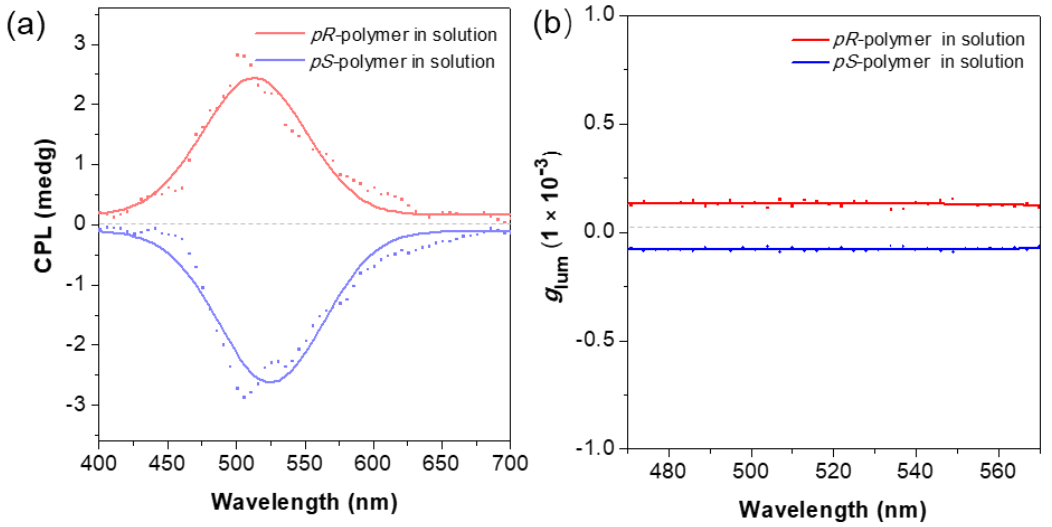

Figure S23. (a) The CPL spectra and (b) the g_{lum} spectra of *pR*-polymer and *pS*-polymer in solutions. Solution concentration: 10 μM. The scattered data represents the raw data and the solid lines are the fitting curve obtained by the Gaussian algorithm.

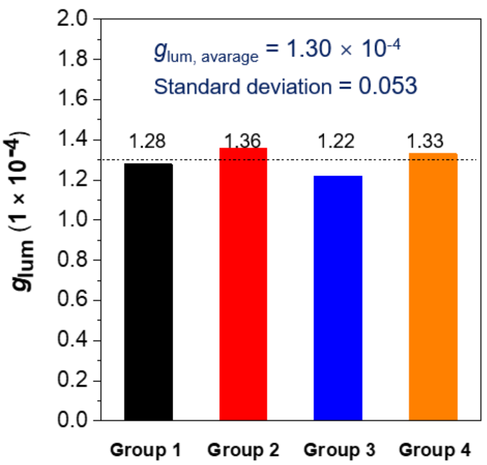

Figure S24. The g_{lum} value of *pR*-polymer in solutions measured for different times. Group 1, group 2, group 3 and group 4 represents the sample used for the measurement at each time under the same conditions. Solution concentration: 10 μM.

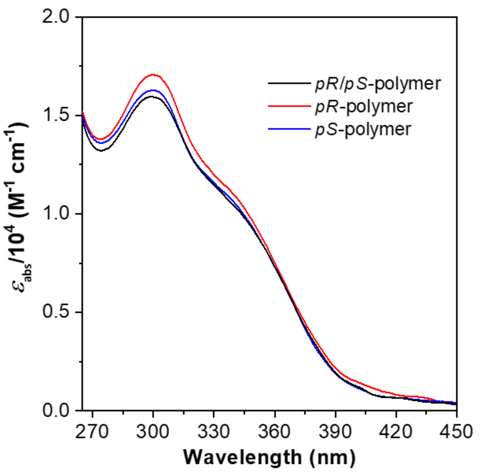

Figure S25. The UV-vis absorption spectra of *pR*-polymer, *pS*-polymer and *pR*/*pS*-polymer in DMSO/water mixture with a water content of 90% measured with an integrating sphere. Concentration: 10 μM.

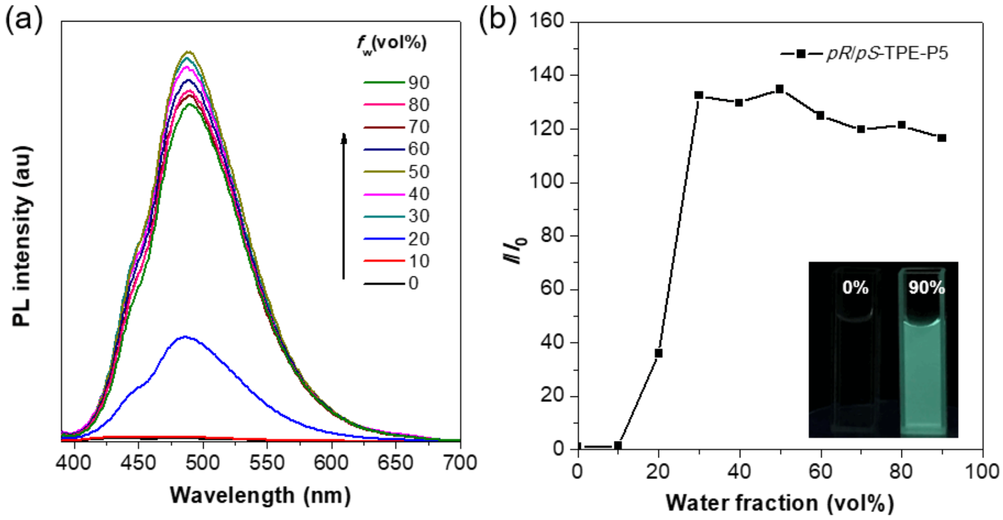

Figure S26. (a) PL spectra of *pR*/*pS*-TPE-P5 in DMSO/water mixtures with different water fractions (f_w). Concentration: 10 μM. Excitation wavelength = 298 nm. (b) Plot of the relative PL intensity (I/I_0) values of *pR*/*pS*-TPE-P5 versus the water fraction. Inset: fluorescent photographs of *pR*/*pS*-TPE-P5 in pure DMSO solution and DMSO/water mixture with a f_w of 90% taken under 365 nm UV irradiation.

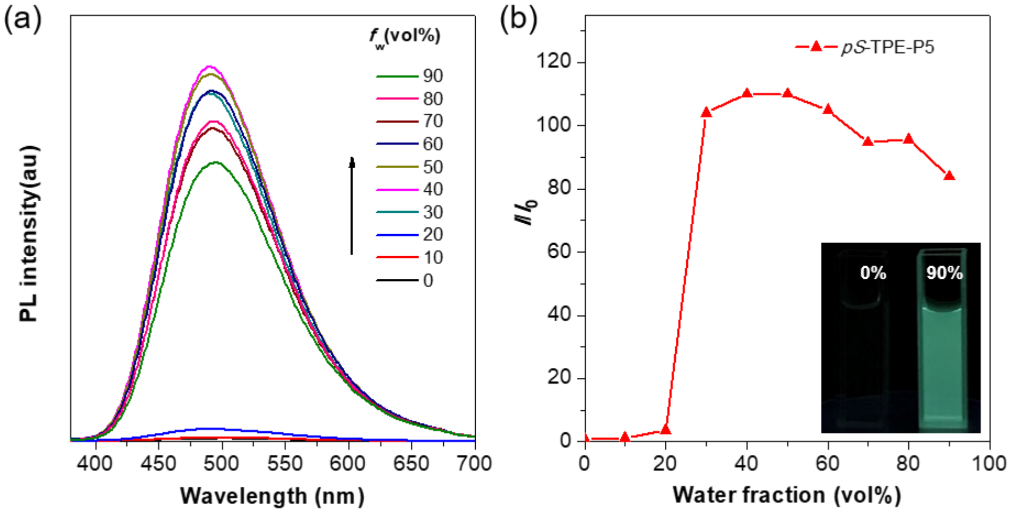

Figure S27. (a) PL spectra of *pS*-TPE-P5 in DMSO/water mixtures with different water fractions (f_w). Concentration: 10 μM. Excitation wavelength = 298 nm. (b) Plot of the relative PL intensity (I/I_0) values of *pS*-TPE-P5 versus the water fraction. Inset: fluorescent photographs of *pS*-TPE-P5 in pure DMSO solution and DMSO/water mixture with a f_w of 90% taken under 365 nm UV irradiation.

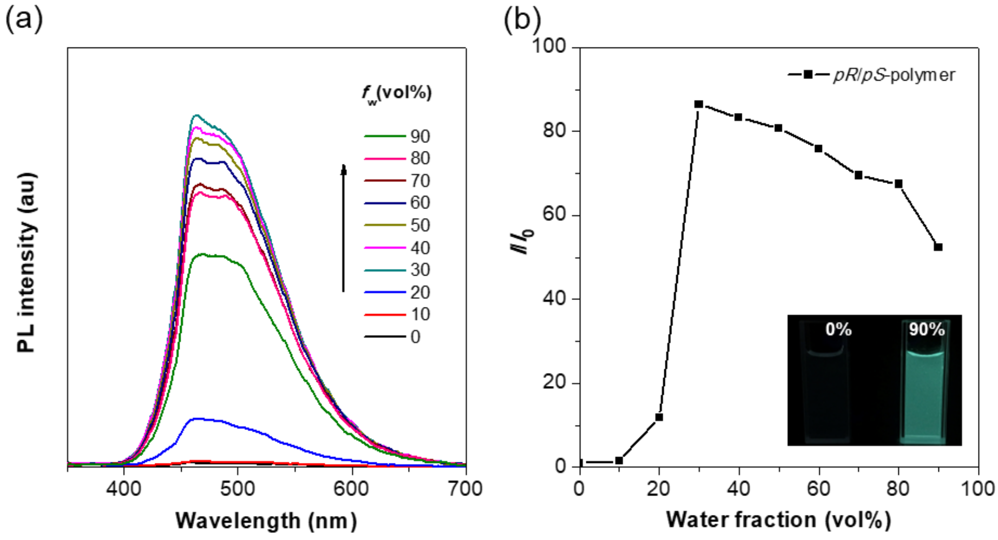

Figure S28. (a) PL spectra of *pR/pS*-polymer in DMSO/water mixtures with different water fractions (f_w). Concentration: 10 μM. Excitation wavelength = 298 nm. (b) Plot of the relative PL intensity (I/I_0) values of *pR/pS*-polymer versus the water fraction. Inset: fluorescent photographs of *pR/pS*-polymer in pure DMSO solution and DMSO/water mixture with a f_w of 90% taken under 365 nm UV irradiation.

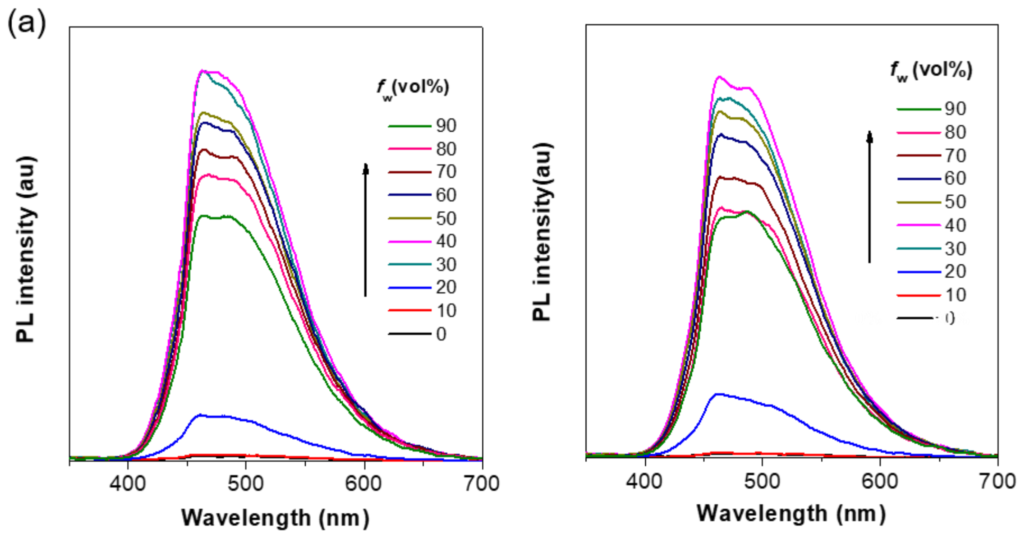

Figure S29. PL spectra of (a) *pS*-polymer and (b) *pR*-polymer in DMSO/water mixtures with different water fractions (f_w). Concentration: 10 μM. Excitation wavelength = 298 nm.

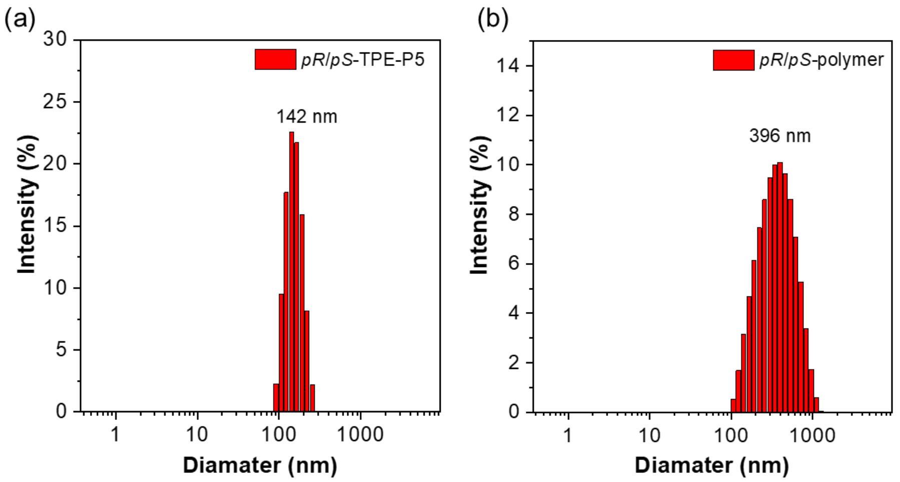

Figure S30. The particle size distributions of (a) *pR*/*pS*-TPE-P5 and (b) *pR*/*pS*-polymer in 90% H_2O measured by DLS. Concentration: 10 μM.

Table S2 Statistical table of fluorescence lifetime (τ)

Parameter	TPE-P5 in solution[a]			Polymer in solution		
	pR	*pS*	*pR/pS*	*pR*	*pS*	*pR/pS*
τ (ns)	0.27	0.29	0.26	0.36	0.35	0.35
**Parameter**	**TPE-P5 aggregates[b]**			**Polymer aggregates**		
	pR	*pS*	*pR/pS*	*pR*	*pS*	*pR/pS*
τ (ns)	3.09	2.98	2.92	3.71	3.71	3.69

[a] Solvent: DMSO. Concentration: 10 μM.

[b] Solvent: H_2O/DMSO = 90/10. Concentration: 10 μM.

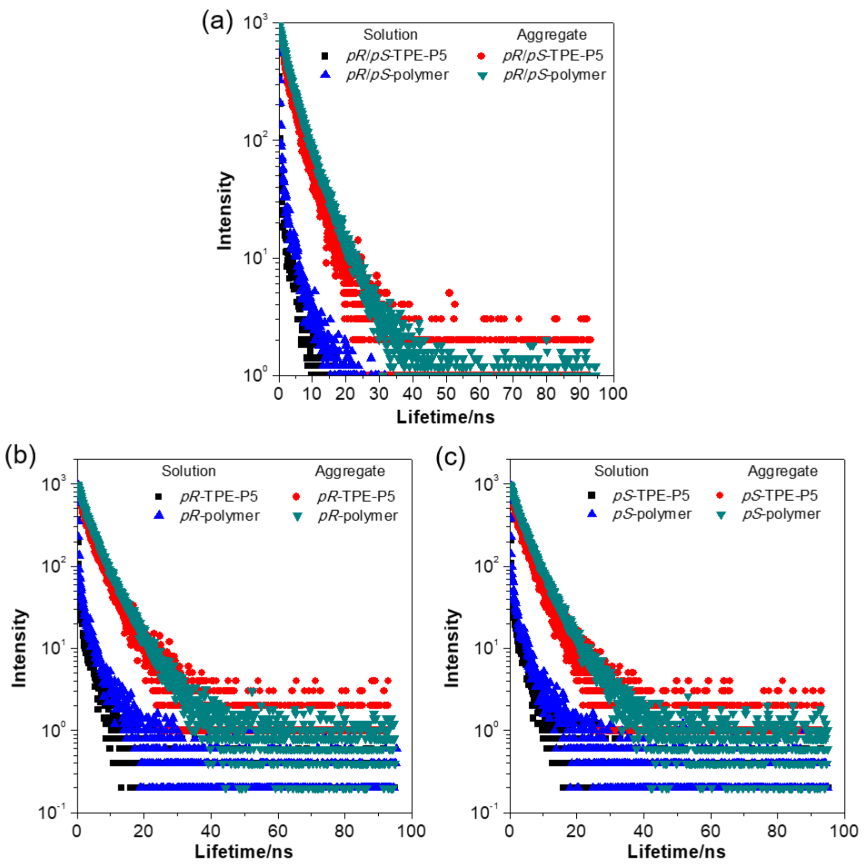

Figure S31. (a) Fluorescence lifetime of the DMSO solution of *pR*/*pS*-TPE-P5, the DMSO solution of *pR*/*pS*-polymer, *pR*/*pS*-TPE-P5 aggregates in the DMSO/water mixture with 90% water and *pR*/*pS*-polymer aggregates in the DMSO/water mixture with 90% water. (b) Fluorescence lifetime of the DMSO solution of *pR*-TPE-P5, the DMSO solution of *pR*-polymer, *pR*-TPE-P5 aggregates in the DMSO/water mixture with 90% water and *pR*-polymer aggregates in the DMSO/water mixture with 90% water. (c) Fluorescence lifetime of the DMSO solution of *pS*-TPE-P5, the DMSO solution of *pS*-polymer, *pS*-TPE-P5 aggregates in the DMSO/water mixture with 90% H_2O and *pS*-polymer aggregates in the DMSO/water mixture with 90% water. Concentration adopted for the measurements is 10 μM.

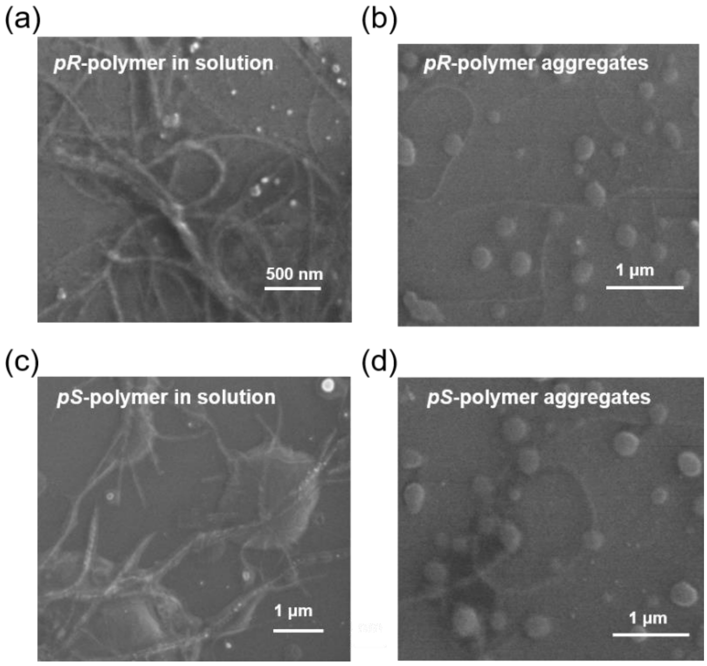

Figure S32. (a and c) SEM images of (a) *pR*-polymer and (c) *pS*-polymer samples prepared in solutions. (b and d) SEM images of (b) *pR*-polymer and (d) *pS-polymer* aggregates in in aggregate states (f_w = 90%) with 90% water. Solution concentration: 1 μM.

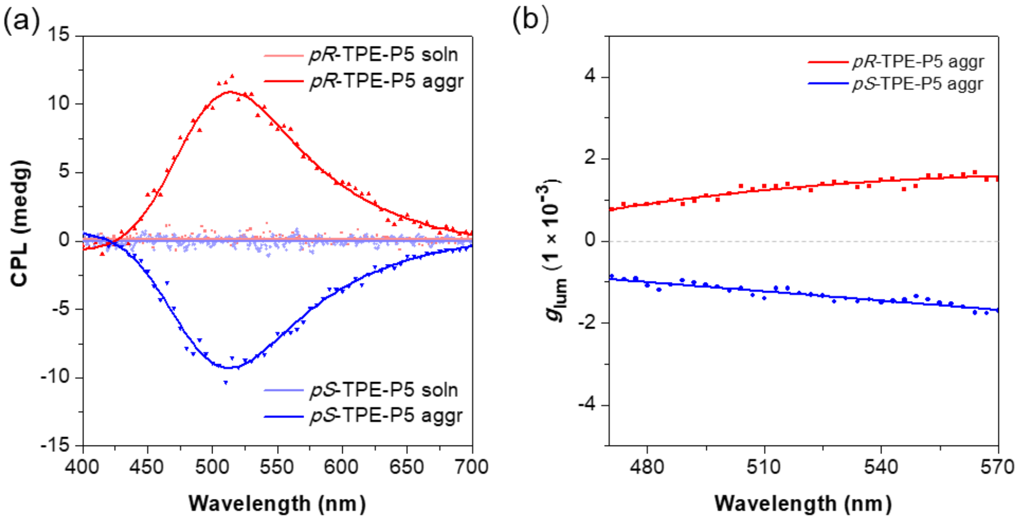

Figure S33. (a) The CPL spectra of *pR*-TPE-P5 and *pS*-TPE-P5 in solution and aggregate states (f_w = 90%). (b) The g_{lum} spectra of *pR*-TPE-P5 and *pS*-TPE-P5 aggregates in aggregate states with 90% water. Solution concentration: 10 μM. The scattered data represents the raw data and the solid lines are the fitting curves obtained by the Gaussian algorithm.

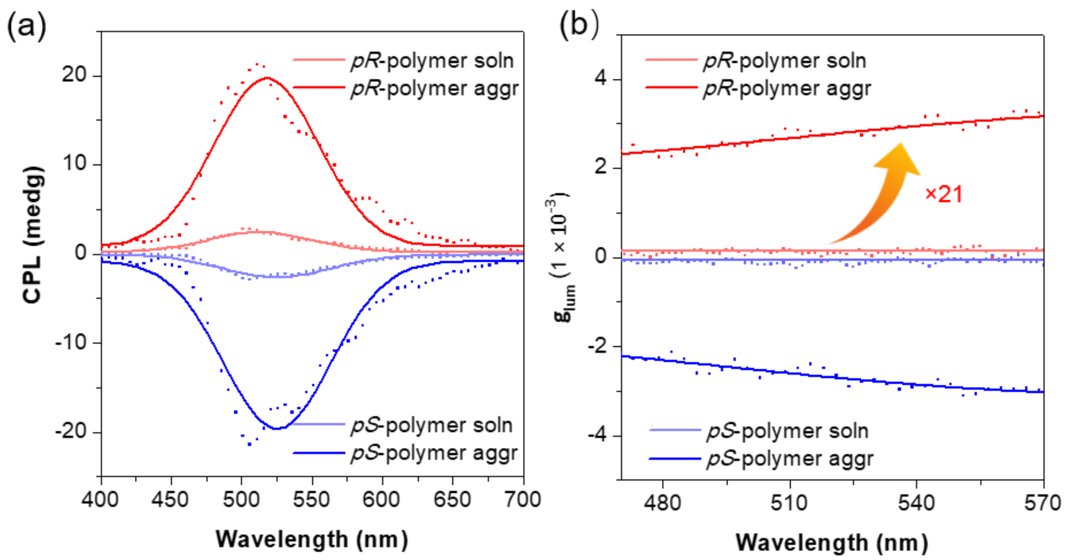

Figure S34. (a) The CPL spectra of *pR*-polymer and *pS*-polymer in solution and aggregate states (f_w = 90%). (b) The g_{lum} spectra of *pR*-polymer and *pS*-polymer aggregates in solution and aggregate states (f_w = 90%). Solution concentration: 10 μM. The scattered data represents the raw data and the solid lines are the fitting curves obtained by the Gaussian algorithm.

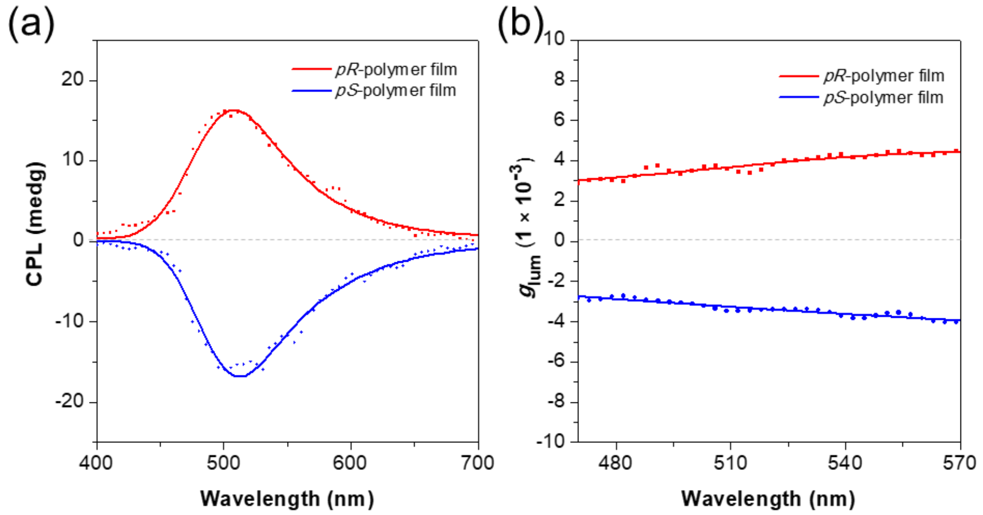

Figure S35. (a) The CPL spectra and (b) the g_{lum} spectra of *pR*-polymer film and *pS*-polymer film. The scattered data represents the raw data and the solid lines are the fitting curves obtained by the Gaussian algorithm. Films for the measurement were prepared by drop-casting the DCE solution of the polymers (concentration: 0.5 wt%) onto quartz plates.

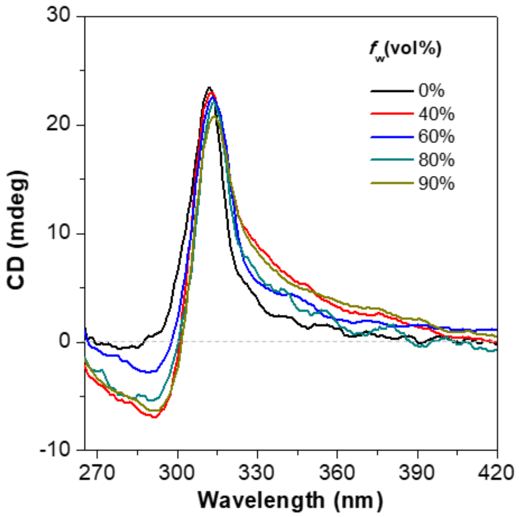

Figure S36. CD spectra of *pR*-polymer in solution state (10 μM) and aggregate state in aqueous media with different water fractions.

To provide an immediate and integrated view of the main photophysical parameters determining the total amount of circularly polarized photons emitted by a material, a parameter of brightness for CPL (B_{CPL}) was recently proposed following the concept of fluorescence brightness, and the value of B_{CPL} is defined by the following equation.[4,5]

$$B_{CPL} = \varepsilon_{abs} \times \Phi \times |g_{lum}|/2$$

where ε_{abs} denotes the molar extinction coefficient of the sample at the maximum absorption wavelength measured with an integrating sphere (Figure S24), Φ denotes the fluorescence quantum yield, and $|g_{lum}|$ denotes the luminescence dissymmetry factor.

5. Theoretical Calculations of the Singlet Excited States

Time-dependent DFT (TD-DFT) calculations on the geometrical properties and electronic properties of singlet excited states of *pR*-TPE-P5, *pS*-TPE-P5 and *pR*-TPE-P5 dimer were performed at the wB97XD/Def2-SVP level using the Gaussian 16 program package.[3] The optimized S_1 geometries of the dimer was obtained by conditionally optimizing the S_1 configuration of *pR*-TPE-P5. The CPL-relevant parameters, including the electric transition dipole moment (μ), the magnetic transition dipole moment (m), and the vector angle between μ and m ($\theta_{\mu,m}$) were calculated from the optimized S_1 geometries of the monomers and the dimer during the $S_1 \rightarrow S_0$ transitions. The calculated dissymmetry factor (g_{lum}) of CPL is estimated according to the following formula[6]:

$$g_{\mathrm{lum}} = \frac{4|m|\cos\theta_{\mu,m}}{|\mu|}$$

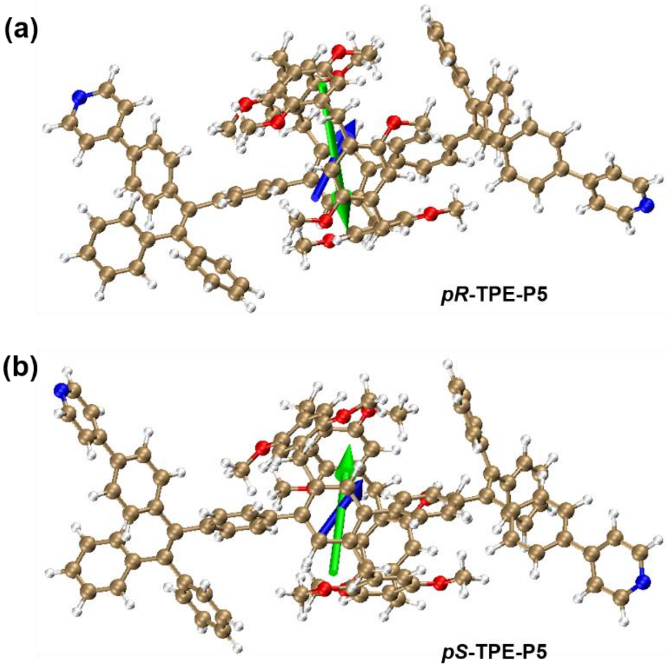

Figure S37. Transition dipole moments of (a) pR-TPE-P5 and (b) pS-TPE-P5 for the $S_1 \rightarrow S_0$ transition. The electric transition dipole moment vectors (μ) are shown in green, and the magnetic transition dipole moment vectors (m) are shown in blue. The length of the μ vector is amplified 4 times for charity.

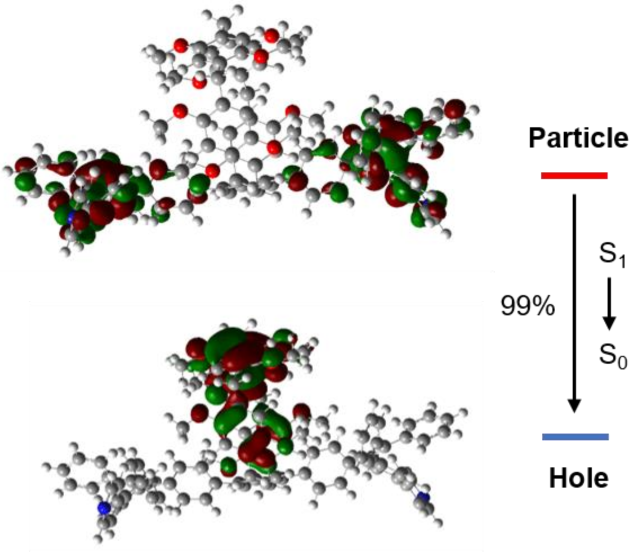

pS-TPE-P5

Figure S38. Natural transition orbitals of *pS*-TPE-P5 calculated with the optimized S_1 geometry by TD-DFT at the wB97XD/def2-SVP level. The percentage refers to the proportion of the dominant particle-to-hole transition.

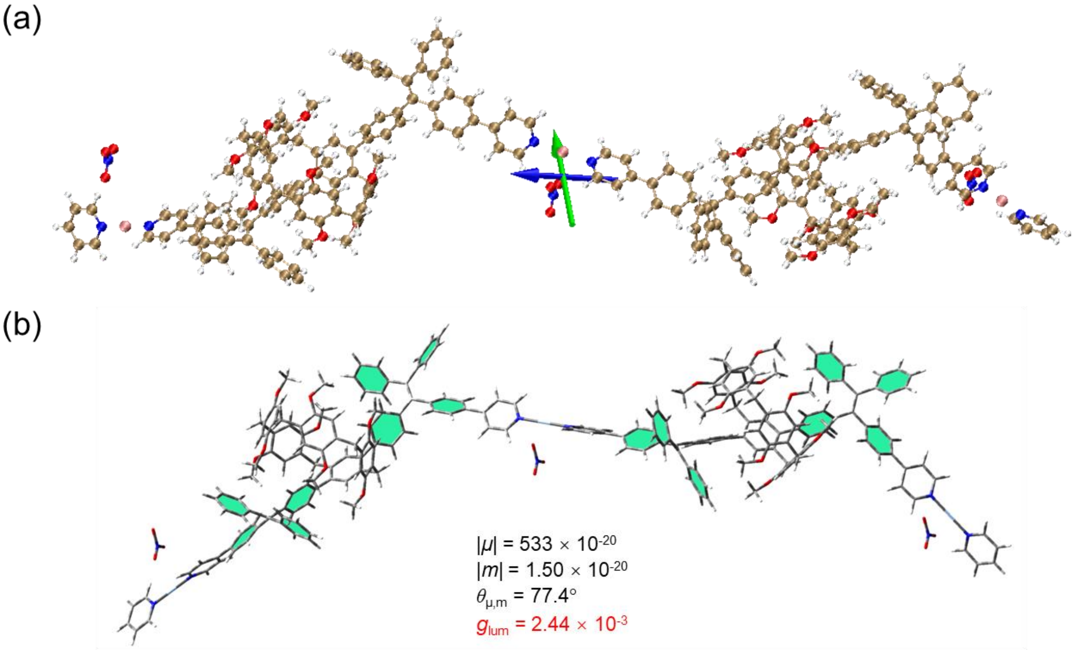

Figure S39. (a) Transition dipole moments of *pR*-TPE-P5 dimer for the $S_1 \rightarrow S_0$ transition. The electric transition dipole moment vectors (μ) are shown in green, and the magnetic transition dipole moment vectors (m) are shown in blue. The length of the μ and m vector is amplified 5 times for charity. (b) The optimized S_1 geometries of *pR*-TPE-P5 dimer obtained by conditionally optimizing the S_1 configuration of pR-TPE-P5 and its calculated transition dipole moments for the $S_1 \rightarrow S_0$ transition. The unit for $|\mu|$ and $|m|$ is esu cm and erg G^{-1}, respectively.

Reference

(1) Zhu, H.; Li, Q.; Shi, B.; Xing, H.; Sun, Y.; Lu, S.; Shangguan, L.; Li, X.; Huang, F.; Stang, P. J., Formation of Planar Chiral Platinum Triangles via Pillar[5]arene for Circularly Polarized Luminescence. *J. Am. Chem. Soc.* **2020,** *142*, 17340-17345.

(2) Chen, J.-F.; Yin, X.; Wang, B.; Zhang, K.; Meng, G.; Zhang, S.; Shi, Y.; Wang, N.; Wang, S.; Chen, P., Planar Chiral Organoboranes with Thermoresponsive Emission and Circularly Polarized Luminescence: Integration of Pillar[5]arenes with Boron Chemistry. *Angew. Chem. Int. Ed.* **2020,** *59*, 11267-11272.

(3) Frisch, M. J.; Trucks, G. W.; Schlegel, H. B.; Scuseria, G. E.; Robb, M. A.; Cheeseman, J. R.; Scalmani, G.; Barone, V.; Petersson, G. A.; Nakatsuji, H.; Li, X.; Caricato, M.; Marenich, A. V.; Bloino, J.; Janesko, B. G.; Gomperts, R.; Mennucci, B.; Hratchian, H. P.; Ortiz, J. V.; Izmaylov, A. F.; Sonnenberg, J. L.; Williams-Young, D.; Ding, F.; Lipparini, F.; Egidi, F.; Goings, J.; Peng, B.; Petrone, A.; Henderson, T.; Ranasinghe, D.; Zakrzewski, V. G.; Gao, J.; Rega, N.; Zheng, G.; Liang, W.; Hada, M.; Ehara, M.; Toyota, K.; Fukuda, R.; Hasegawa, J.; Ishida, M.; Nakajima, T.; Honda, Y.; Kitao, O.; Nakai, H.; Vreven, T.; Throssell, K.; Montgomery, J. A.; Jr., Peralta, J. E.; Ogliaro, F.; Bearpark, M. J.; Heyd, J. J.; Brothers, E. N.; Kudin, K. N.; Staroverov, V. N.; Keith, T. A.; Kobayashi, R.; Normand, J.; Raghavachari, K.; Rendell, A. P.; Burant, J. C.; Iyengar, S. S.; Tomasi, J.; Cossi, M.; Millam, J. M.; Klene, M.; Adamo, C.; Cammi, R.; Ochterski, J. W.; Martin, R. L.; Morokuma, K.; Farkas, O.; Foresman, J. B.; and Fox, D. J. Gaussian, Inc., Gaussian 16, Revision C.01, Wallingford CT, **2019**.

(4) Arrico, L.; Di Bari, L.; Zinna, F., Quantifying the Overall Efficiency of Circularly Polarized Emitters. *Chem. Eur. J.* **2021,** *27*, 2920-2934.

(5) Wong, K.-L.; Bünzli, J.-C. G.; Tanner, P. A., Quantum yield and brightness. *Journal of Luminescence* **2020,** *224*, 117256.

(6) Ying, A.; Ai, Y.; Yang, C.; Gong, S., Aggregation-Dependent Circularly Polarized Luminescence and Thermally Activated Delayed Fluorescence from Chiral Carbene-CuI-Amide Enantiomers. *Angew. Chem. Int. Ed.* **2022,** *61*, e202210490.
